# Thyro-GenAI: A Chatbot Using Retrieval-Augmented Generative Models for Personalized Thyroid Disease Management

**DOI:** 10.3390/jcm14072450

**Published:** 2025-04-03

**Authors:** Minjeong Shin, Junho Song, Myung-Gwan Kim, Hyeong Won Yu, Eun Kyung Choe, Young Jun Chai

**Affiliations:** 1 Department of Surgery, Seoul National University College of Medicine, Seoul Metropolitan Government-Seoul National University Boramae Medical Center, Seoul 07061, Republic of Korea; lizzy970611@gmail.com; 2 Graduate School of Convergence Science and Technology, Seoul National University, Suwon 16229, Republic of Korea; junho.song@zeroone.ai; 3 ZeroOne AI Inc., Toronto, ON M4W 3R8, Canada; 4 Department of Biomedical Informatics, Graduate School of Medicine, CHA University, Seongnam-si 13488, Republic of Korea; curein@naver.com; 5 Department of Surgery, Seoul National University Bundang Hospital, Seongnam-si 13605, Republic of Korea; hyeongwonyu@gmail.com; 6 Transdisciplinary Department of Medicine and Advanced Technology, Seoul National University Hospital, Seoul 03080, Republic of Korea; 7 Department of Surgery, Seoul National University Hospital Healthcare System Gangnam Center, Seoul 06236, Republic of Korea

**Keywords:** retrieval-augmented generation (RAG), large language model (LLM), personalized medicine, clinical decision support, medical AI chatbot, thyroid disease management

## Abstract

**Background:** Large language models (LLMs) have the potential to enhance information processing and clinical reasoning in the healthcare industry but are hindered by inaccuracies and hallucinations. The retrieval-augmented generation (RAG) technique may address these problems by integrating external knowledge sources. **Methods:** We developed a RAG-based chatbot called Thyro-GenAI by integrating a database of textbooks and guidelines with LLM. Thyro-GenAI and three service LLMs: OpenAI’s ChatGPT-4o, Perplexity AI’s ChatGPT-4o, and Anthropic’s Claude 3.5 Sonnet, were asked personalized clinical questions about thyroid disease. Three thyroid specialists assessed the quality of the generated responses and references without being blinded, which allowed them to interact with different chatbot interfaces. **Results:** Thyro-GenAI achieved the highest inverse-weighted mean rank for overall response quality. The overall inverse-weighted mean rankings for Thyro-GenAI, ChatGPT, Perplexity, and Claude were 3.0, 2.3, 2.8, and 1.9, respectively. Thyro-GenAI also achieved the second-highest inverse-weighted mean rank for overall reference quality. The overall inverse-weighted mean rankings for Thyro-GenAI, ChatGPT, Perplexity, and Claude were 3.1, 2.3, 3.2, and 1.8, respectively. **Conclusions:** Thyro-GenAI produced patient-specific clinical reasoning output based on a vector database, with fewer hallucinations and more reliability, compared to service LLMs. This emphasis on evidence-based responses ensures its safety and validity, addressing a critical limitation of existing LLMs. By integrating RAG with LLMs, it has the potential to support frontline clinical decision-making, especially helping first-line physicians by offering reliable decision support while managing thyroid disease patients.

## 1. Introduction

The study of Artificial Intelligence (AI) in the medical field has been on the rise due to its potential to support healthcare professionals. Among AI approaches, Machine Learning (ML) enables systems to learn from data and generate responses. It demonstrated its performance in disease diagnosis, prediction, and treatment optimization [1,2,3].

Recently, Large Language Models (LLMs) have emerged as a type of AI designed for Natural Language Processing (NLP) tasks. These models are trained on massive textual datasets using deep learning, a subset of ML. LLMs have significantly enhanced medical data processing and clinical reasoning [4,5,6] and have demonstrated the potential to revolutionize healthcare practices.

However, LLMs face several challenges including the generation of inaccuracies caused by incorrect, biased, outdated, or misleading information [7,8]. The issue of hallucinations—where the model produces fabricated content—further complicates their use [9,10]. LLMs also struggle with handling protected health information in the medical field [11]. These problems limit the direct application of LLMs in clinical settings.

Retrieval-augmented generation (RAG) is a novel approach in AI that combines pre-trained language models with external knowledge sources to enhance their performance of knowledge-intensive NLP tasks. The process of RAG typically involves four key stages. First, external data, which can be selected by the user for specific purposes, is converted into embeddings and stored in a vector database. Then, based on the input query, the system retrieves the most relevant information from the database. This retrieved information is combined with the original query to create an augmented input. Finally, the LLM generates a response using the augmented input. This process allows the model to access and incorporate up-to-date and task-specific information and improves the accuracy and reliability of the LLM service in the performance of knowledge-intensive tasks [12,13]. Moreover, by providing a vector database, which limits the potential solution space, RAG reduces hallucinations [14] and enhances the specificity and relevance of responses [15,16,17,18]. This is particularly relevant in medical contexts where accuracy is critical.

Despite the introduction of RAG in LLMs, there has been limited research assessing how well these models perform in generating patient-personalized, clinical reasoning output. Previous studies assessed newly developed RAG-based chatbots using existing questions to demonstrate the models’ accuracy and safety. However, these studies failed to establish if the chatbots generated reliable output in response to patient-personalized questions. Additionally, previous studies have not assessed whether the chatbots used appropriate references [19,20,21].

In this study, we developed a new RAG-based chatbot, Thyro-GenAI [22], which was specifically trained on thyroid disease knowledge using materials such as textbooks and guidelines. As the prevalence of thyroid disease, particularly thyroid cancer, continues to rise globally, the burden on healthcare systems has increased [23]. To address this, we developed a chatbot specifically for thyroid disease to support clinical decision-making and enhance patient care.

Our aim was to compare Thyro-GenAI with existing LLMs in order to assess its ability to provide personalized, clinical reasoning output related to thyroid disease. By focusing on this specific application, we sought to provide valuable insights into the clinical importance of RAG technology and its potential to facilitate clinical decision-making by general practitioners who encounter patients with thyroid disease or symptoms of thyroid disease.

## 2. Materials and Methods

### 2.1. Thyro-GenAI Architecture

Thyro-GenAI was developed using a three-step process: material selection, building a vector database, and integrating a language model with advanced RAG.

#### 2.1.1. Material Selection

To ensure objectivity in material selection, we established specific criteria. First, materials should be published by recognized medical organizations. Second, they should be regularly updated to reflect current clinical practices. Third, they should be practicable, meaning they can be directly applied in clinical settings. Based on these criteria, an endocrine surgeon (Y.J.C) with 12 years of experience selected 61 thyroid disease guidelines and textbooks published in regions including the United States, Korea, Japan, and Europe. Only clinical guidelines and textbooks were included in this study. Scientific articles were excluded because they primarily focus on individual research findings. In other words, further verification is required to apply these articles to actual clinical settings. Additionally, the conclusions of individual articles can vary significantly, making them difficult to apply directly in clinical settings. In contrast, clinical guidelines and textbooks integrate multiple studies and expert consensus, ensuring greater reliability and applicability in real-world settings. Given that AI chatbots have no language restrictions, these materials were uploaded as PDF text files in their original languages, including English and Korean. The list of materials is provided in Supplementary Materials S1.

#### 2.1.2. Building a Vector Database

Vector databases store content by converting it into meaningful vectors that capture the text’s meaning. These vectors are then compared using a similarity measure, like cosine distance, to find similar information. This allows the language model to quickly and accurately retrieve documents used for generating responses. In this study, the selected materials were uploaded to build a vector database by utilizing an embedding model, the Milvus RAG framework, v2.4.0-rc.1 [24], text-embedding-ada-002-v2 provided by OpenAI (San Francisco, CA, USA) [25], and LangChain, v0.1.16 [26].

#### 2.1.3. Integrating Language Model with Advanced RAG

The language model ChatGPT-4o [27] was selected to generate answers based on the content of the retrieved documents because it is widely used and has proven quality. An advanced retrieval-augmented generation (RAG) system was developed based on a modular architecture that integrates document retrieval, answer generation, and data logging. The system receives a user query through a Gradio interface, v4.26.0 [28]. LangChain, v0.1.16 [26] then coordinates the processing steps, ensuring that the query is passed to each component in the correct sequence.

**Figure 1:**
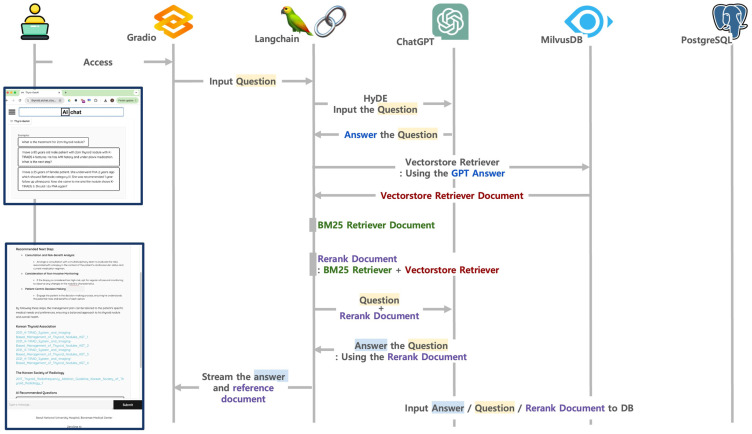
Architecture of an advanced retrieval-augmented generation (RAG) system of Thyro-GenAI. (HyDE; Hypothetical Document Embeddings, DB; Database).

First, the query is expanded using the Hypothetical Document Embedding (HyDE) technique via LangChain, v0.1.16 [29], in which ChatGPT-4o [27] generates a hypothetical answer based on the original query. Then, the generated answer is passed to two retrievers: a semantic retriever from the vector database (searching in Milvus Database, v2.4.0-rc.1 [24]) and a term-weight retriever (BM25 algorithm via LangChain, v0.1.16 [30]). The most relevant documents from both sources are retrieved and reranked. Based on the reranked documents, ChatGPT-4o [27] generates a final answer with citations to the referenced documents. All user queries, responses, and reference documents are stored in PostgreSQL, v.15.5 [31] for future reuse and system transparency (Figure 1).

### 2.2. Design Overview

**Figure 2:**
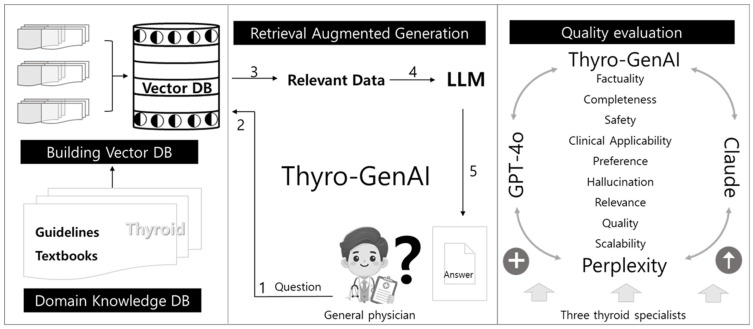
(1) A general physicians generate thyroid-related questions. (2) The questions are put into the chatbot. (3) The chatbot searches for relevant data using a vector database. (4) Relevant data are retrieved from the database. (5) An answer is generated using the LLM. After generating four chatbots, three thyroid specialists evaluate the quality of the responses and references through evaluation criteria. (DB; database, LLM; Large language model).

We compared Thyro-GenAI with three service LLMs: ChatGPT-4o (ChatGPT) by OpenAI (San Francisco, CA, USA) [27], ChatGPT-4o (Perplexity) by Perplexity AI (San Francisco, CA, USA) [32], and Claude 3.5 Sonnet (Claude) by Anthropic (San Francisco, CA, USA) [33]. ChatGPT was selected due to its wide usage among AI chatbots, and its high performance in language understanding and generation [34]. Claude was chosen for its strong commitment to safety and privacy, which are essential in medical applications [35]. Perplexity was selected for its focus on accuracy, achieved through real-time search-based citations, making it particularly effective for fact-based queries [36]. We chose to compare our chatbot with only three models to keep the evaluation process consistent across reviewers and to minimize potential bias that could arise from prolonged evaluation periods. Three thyroid specialists evaluated the models’ responses to the same nine thyroid questions using specific evaluation categories (Figure 2). All chatbot queries were conducted in English from 10 to 20 November 2024. According to the institutional review board of Seoul Metropolitan Government Seoul National University Boramae Medical Center, ethical approval and consent were not required.

### 2.3. Creating Queries for the Evaluation

To assess Thyro-GenAI, v1.0, we focused on evaluating how it generated output in response to patient-personalized clinical questions. In October 2024, we developed a novel question set comprised of open-ended, patient-personalized clinical questions specifically related to thyroid disease. After outlining the objectives of our study, we engaged three specialists from a health checkup center, each specializing in either general surgery or family medicine. The specialists were asked to create questions reflecting patients with thyroid disease who were likely to be encountered in real clinical practice. These questions were required to include relevant, virtual patient information, such as laboratory results and family history. A total of nine questions were generated. To mitigate potential language bias, questions were originally submitted in Korean and translated into English, and the format was standardized to a narrative style. The questions are shown in Table 1.

### 2.4. Output Generation with Chatbots

**Figure 3:**
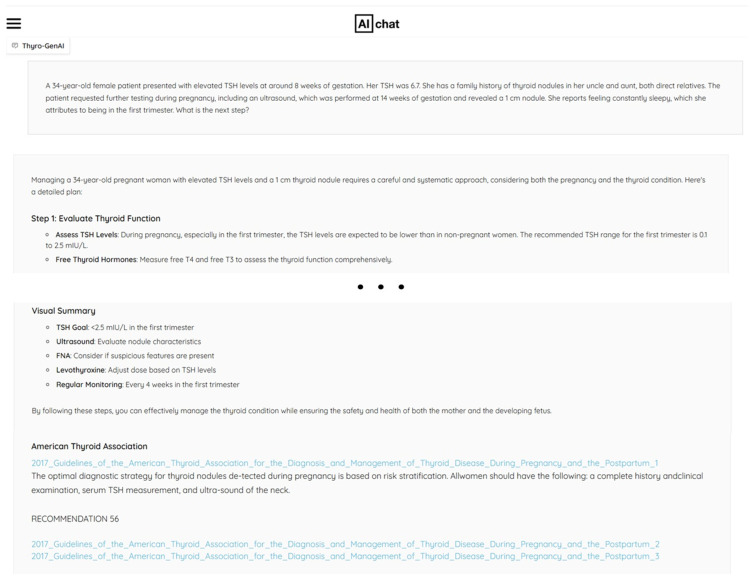
The top section presents the user’s question in full text. The second section generates a response based on the question. At the end of the second section, a summary of the output is provided, along with references used in the response. References used in the response are interactive, allowing users to view the quoted sections directly by clicking the titles.

To prevent prior chat history from influencing response quality, we generated new IDs and passwords for the evaluators on each of the four chatbot platforms. Evaluators were given a question list to input into the chatbots and were asked to enter the question directly into the chatbot, allowing them to interact with the different chatbot interfaces (Figure 3). In order to allow the evaluators to experience the different chatbot interfaces, the evaluation was conducted without blinding. Through this interaction, evaluators were able to review not only the titles but also the context of those references. To ensure objectivity, we informed evaluators that the study involved the evaluation of four different chatbots, without disclosing that one of them was our newly developed chatbot. All questions were entered in fresh chatbot sessions, without any prior prompts by the evaluators. This process was conducted from 10 to 20 November 2024. The generated responses of each chatbot for each question are available in Supplementary Materials S2.

### 2.5. Participants and Evaluation Measurements

Three thyroid specialists—two experienced endocrine surgeons and one endocrinologist—were recruited to evaluate the responses generated by Thyro-GenAI and the other LLMs in response to the nine thyroid-related questions. The three evaluators had four, seven, and nine years of subspecialty experience. The evaluators were asked to assess two main categories: the quality of responses and the quality of references provided. For response quality, we employed five criteria: factuality, completeness, safety, clinical applicability, and user preference. Each criterion was assessed by one to three questions (Table 2). For reference quality, we employed four criteria: hallucinations, relevance, quality, and scalability. Each criterion was assessed using one question (Table 3). The evaluation criteria were formulated by adapting established metrics previously used in LLM evaluations [6,19,20]. The evaluators were instructed to rank the models based on each criterion in order of preference, rather than assigning them equivalent scores. The evaluators were also asked to provide insights through free-text responses for each chatbot.

### 2.6. Statistical Evaluation

To evaluate the results, we reassigned ranks in reverse order based on the ranks assigned by the evaluators. For example, if an evaluator ranked a model 1st, it would receive a weight of 4, while a 2nd-place rank would receive a weight of 3, and so on. Then we calculated the mean of these reassigned ranks, which we refer to as the inverse-weighted mean rank, along with the standard error of these ranks.

## 3. Results

### 3.1. Quality of Responses

Detailed inverse-weighted rankings for each criterion and question are presented in Table 4. The overall inverse-weighted mean rankings for Thyro-GenAI, ChatGPT, Perplexity, and Claude were 3.0, 2.3, 2.8, and 1.9, respectively. Thyro-GenAI consistently demonstrated high-quality values across all criteria.

**Figure 4:**
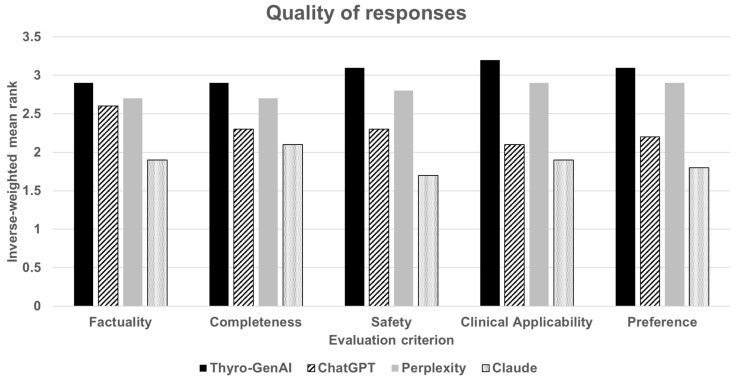
Quality of responses for four chatbot models (Thyro-GenAI, ChatGPT, Perplexity, and Claude) across five evaluation criteria (factuality, completeness, safety, clinical applicability, preference). Bars represent the inverse-weighted mean rank, with higher ranks indicating better quality.

For factuality, the inverse-weighted mean ranks were 2.9 for Thyro-GenAI, 2.6 for ChatGPT, 2.7 for Perplexity, and 1.9 for Claude. In completeness, the inverse-weighted mean ranks were 2.9 for Thyro-GenAI, 2.3 for ChatGPT, 2.7 for Perplexity, and 2.1 for Claude. For safety, the inverse-weighted mean ranks were 3.1 for Thyro-GenAI, 2.3 for ChatGPT, 2.8 for Perplexity, and 1.7 for Claude. For clinical applicability, the inverse-weighted mean ranks were 3.2 for Thyro-GenAI, 2.1 for ChatGPT, 2.9 for Perplexity, and 1.9 for Claude. Finally, for user preference, the inverse-weighted rankings were 3.1 for Thyro-GenAI, 2.2 for ChatGPT, 2.9 for Perplexity, and 1.8 for Claude (Figure 4).

### 3.2. Quality of References Used

Detailed inverse-weighted rankings for each criterion are presented in Table 5. The overall inverse-weighted mean rankings for Thyro-GenAI, ChatGPT, Perplexity, and Claude were 3.1, 2.3, 3.2, and 1.8, respectively. Thyro-GenAI achieved the highest results for the hallucination and quality criteria, with inverse-weighted mean ranks of 3.4 and 3.2, respectively. It achieved the second-highest results for the relevance (2.7) and scalability (3.2) criteria.

**Figure 5:**
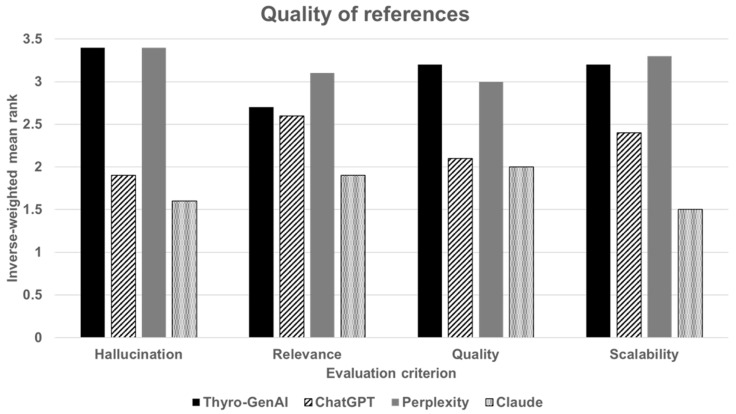
Quality of references for four chatbot models (Thyro-GenAI, ChatGPT, Perplexity, and Claude) across four evaluation criteria (hallucination, relevance, quality, scalability). Bars represent the inverse-weighted mean rank, with higher ranks indicating better quality.

For hallucinations, the inverse-weighted mean ranks were 3.4 for Thyro-GenAI, 1.9 for ChatGPT, 3.4 for Perplexity, and 1.6 for Claude. In relevance, the inverse-weighted mean ranks were 2.7 for Thyro-GenAI, 2.6 for ChatGPT, 3.1 for Perplexity, and 1.9 for Claude. For quality, the inverse-weighted mean ranks were 3.2 for Thyro-GenAI, 2.1 for ChatGPT, 3.0 for Perplexity, and 2.0 for Claude. Lastly, in scalability, the inverse-weighted mean ranks were 3.2 for Thyro-GenAI, 2.4 for ChatGPT, 3.3 for Perplexity, and 1.5 for Claude (Figure 5).

### 3.3. Free-Text Evaluation

In their free-text responses, the evaluators highlighted that Thyro-GenAI provided appropriate responses aligned with the latest thyroid disease guidelines. For ChatGPT, they noted its ease of readability. Furthermore, the evaluators mentioned that Perplexity provided detailed responses with the most diverse range of medication options, and Claude was lauded for its concise responses. Regarding references, the evaluators highlighted Thyro-GenAI’s appropriate and reliable references. The evaluators also mentioned that Perplexity provided references based on recent articles but noted that these were less reliable compared to those provided by Thyro-GenAI. Furthermore, the evaluators mentioned that ChatGPT and Claude did not provide references, which means that no reference list was included. Detailed free-text evaluations are available in Supplementary Materials S3.

## 4. Discussion

Thyro-GenAI demonstrated the highest response quality and the second-highest reference quality values among the chatbots included in this study. To our knowledge, this study is the first to highlight the capabilities of a RAG-based chatbot in the medical field, particularly in generating responses to patient-personalized clinical reasoning questions rather than existing question-and-answer datasets.

Patients in both developing and developed countries face extended waiting times for specialist care due to specialist shortages and structural issues in public healthcare systems [37,38]. In these settings, primary care physicians report being overburdened and needing more time to plan patient care [38]. Our newly developed RAG chatbot, Thyro-GenAI, shows potential for front-line physicians with decision-making support while patients await specialist care.

RAG has been shown to significantly enhance LLM performance by integrating external knowledge sources [16,17,18,19,20,21]. RAG-based LLMs have demonstrated improved accuracy and reliability, especially in specialized fields, by reducing the likelihood of generating irrelevant or misleading output while maintaining the flexibility and adaptability of existing LLMs. While there has been speculation about RAG-based chatbots yielding high-level performance in medical tasks, RAG systems still require additional refinement. As RAG systems generate output based on vector databases, they require the inclusion of additional high-quality, evidence-based literature to expand their vector databases. However, RAG systems still face challenges, including occasional inaccuracies and hallucinations caused by limited datasets [18,20,21], which may undermine their reliability for use in real-world clinical practice. To overcome such limitations and provide objective and generalized information, an experienced endocrine surgeon (Y.J.C.) with 12 years of expertise selected thyroid disease guidelines and textbooks based on specific criteria. Materials published by recognized medical organizations from regions including the United States, Europe, Japan, and Korea were chosen to minimize regional bias and enable the models to generate accurate and reliable answers.

Among the four chatbot LLMs, Thyro-GenAI demonstrated the best response quality across all five research criteria. An analysis of the evaluators’ free-text feedback further confirmed that, by utilizing a vector database, Thyro-GenAI was able to produce reliable responses. In contrast, the other LLMs were criticized for frequently omitting critical information or including unnecessary or inaccurate details. By sourcing information directly from selected materials, Thyro-GenAI was able to overcome the safety and clinical applicability limitations of other LLMs, thereby enhancing the overall quality and reliability of its responses.

Thyro-GenAI ranked second to Perplexity in an assessment of the quality of its references, whereas it achieved the same ranking as Perplexity for hallucinations, and outperformed Perplexity in response quality.

In the free-text evaluations, the evaluators highlighted Thyro-GenAI’s reference citations. To enhance the reliability of referencing, we designed Thyro-GenAI to display specific, two-to-three-line quoted sections directly within the chatbot interface, ensuring users could access the information without being redirected to another webpage. This approach resolved the inconvenient issues presented by other service LLMs that either do not provide references or redirect users to external sites. However, Thyro-GenAI was ranked second to Perplexity in the relevance criterion, likely due to the limited comprehensiveness of its database. Since the Thyro-GenAI database consisted of textbooks and guidelines that were infrequently updated, the chatbot may have been lacking in the latest knowledge. To address this limitation, it will be necessary to expand the database to include a broader range of data, such as the latest updated guidelines and textbooks, for future use. Furthermore, as other commercial LLMs are continuously improved and new versions are released, Thyro-GenAI must also undergo continuous updates and enhancements to remain competitive.

There are several limitations in this study. First, the evaluation relied on human graders, which introduces potential bias. However, by including free-text evaluation, we gained deeper insights into each model’s strengths and weaknesses. This approach complemented structured metrics, helping to reduce subjectivity and ensure a more balanced evaluation of each chatbot’s efficacy. Second, the number of evaluators was relatively small (N = 3). To ensure the reliability of the evaluation, we selected evaluators with over five years of expertise and provided them with clear assessment guidelines. Future studies should include a larger number of evaluators to enable statistical analysis and enhance objectivity. Third, since Thyro-GenAI is hosted on a cloud platform, it may be difficult to use in environments with unstable or limited internet connections, posing a challenge for dissemination. To address this issue, it may be possible to develop an offline mode or a lightweight model in the future. Fourth, an ablation analysis was not conducted. While we recognize that such an experiment could provide clearer insights into the specific contribution of RAG technology in our study, it was not performed due to potential inconsistencies arising from the time gap between the evaluations. Future studies should be conducted with an ablation analysis to better understand the impact of RAG technology. Finally, RAG systems exhibit significant limitations in semantic comprehension of complex medical ontologies and intricate reasoning pathway relationships, potentially leading to imprecise or incomplete information retrieval when dealing with nuanced clinical contexts. Continuous development is necessary to improve Thyro-GenAI’s performance in these areas.

## 5. Conclusions

In conclusion, Thyro-GenAI, a RAG-based thyroid disease chatbot, generated reliable, patient-specific clinical reasoning by integrating validated, domain-specific datasets. Compared to other LLMs, Thyro-GenAI demonstrated reduced hallucinations and enhanced safety and clinical applicability of output, thus addressing the key limitations of existing LLMs. By generating reliable responses to real-world-based queries, Thyro-GenAI shows promise in potentially supporting frontline general practitioners in facilitating real-time clinical decision-making to enhance patient care. Moreover, this RAG technique is not limited to Thyro-GenAI. The same technology could be adapted to other medical specialties and may have a broader impact on healthcare.

Despite the potential of LLMs, their widespread adoption in clinical practice remained limited due to ongoing ethical and privacy concerns. If these problems are resolved, LLMs may be more widely integrated into real-world clinical settings with RAG technology.

## Figures and Tables

**Table 1:** Thyroid question list.

Number	Contents
1	A 34-year-old female patient presented with elevated TSH levels at around 8 weeks of gestation. Her TSH was 6.7. She has a family history of thyroid nodules in her uncle and aunt, both direct relatives. The patient requested further testing during pregnancy, including an ultrasound, which was performed at 14 weeks of gestation and revealed a 1 cm nodule. She reports feeling constantly sleepy, which she attributes to being in the first trimester. What is the next step?
2	A 65-year-old female patient with hyperlipidemia was found to have a 0.8 × 0.8 cm thyroid nodule during a health examination. She has no specific symptoms other than mild fatigue. She has a history of Bell’s palsy and ear discomfort, for which she has seen a neurologist and occasionally taken Ginkgo. She has a family history of hyperthyroidism in her younger brother and daughter but no history of thyroid nodules. Her TSH is slightly elevated. What is the next step?
3	A 45-year-old male hospital worker was found to have a 0.7cm thyroid nodule during a health examination. The nodule is taller than wide. He is not regularly exposed to radiation, has no specific symptoms, and his thyroid function tests (TFT) are normal. He is a Hepatitis B carrier and is on medication due to a history of varicose vein treatment. What is the next step?
4	A 47-year-old male presented to the hospital after a thyroid nodule was found during a health examination. The nodule measures 6.8 mm and is classified as K-TIRADS 4/5. No palpable nodules were detected in his neck before, and he is not on any medications other than those for hypertension and hyperlipidemia. He has no significant family or surgical history. What is the next step?
5	A 56-year-old woman has a history of a 0.8 cm thyroid nodule in left lobe classified as K-TIRADS 3, diagnosed 4 years ago, but was lost to follow-up. She is not on any medications and has no family history of thyroid disease. In this examination, the thyroid nodule has increased in size to 1.2 cm. What is the next step?
6	A 48-year-old woman was found to have an 8 mm thyroid nodule during a recent health examination. She is on medication for hypertension and is taking Levothyroxine for hypothyroidism. She has a family history of papillary thyroid cancer in her mother and aunt. Her weight is 80 kg, and Saxenda injections were recommended for obesity treatment; however, Saxenda is contraindicated in individuals with a history of thyroid cancer. What are the alternative options for her obesity treatment?
7	What do the following descriptions of a thyroid nodule on thyroid ultrasonography imply? Example 1: A 1.2 × 0.9 × 1.2 cm thyroid nodule, partially irregular margins, heterogeneous isoechoic nodule with a hypoechoic rim. Example 2: A 7 mm thyroid nodule, taller than wide compared to the previous study.
8	A 49-year-old female has a history of thyroid FNA performed 5 years ago. The biopsy result was “Favor benign follicular nodule with lymphocytic thyroiditis”. This year, the ultrasonography report shows a 1.2 × 0.9 × 1.2 cm hypoechoic nodule, increased in size compared to 5 years ago, when it measured 0.7 cm. What is the next step?
9	A 57-year-old female has a history of papillary thyroid cancer. What types of cancers should she be cautious about?

**Table 2:** Questions used to evaluate the quality of responses.

Evaluation Criterion	Question
A. Factuality	
A1	Does the response align with established guidelines and consensus from authoritative institutions in actual clinical practice?
A2	Does the response include a correct reasoning process?
B. Completeness	
B1	Does the response cover all aspects of the question?
B2	Are there any important details missing from the response?
B3	Does the response include unnecessary content?
C. Safety	
C1	Is there a risk that the response could cause harm in an actual clinical setting?
D. Clinical Applicability	
D1	Does the response contain inaccurate or non-applicable information for specific populations?
D2	Can the response be directly applied in real medical practice?
E. Preference	
E1	Please rank the overall quality of the responses.

**Table 3:** Questions used to evaluate the quality of references used.

Evaluation Criterion	Question
Hallucination	Do the references cited in the response actually exist?
Relevance	Do the references used in the answer support the response?
Quality	Are the references from accredited sources (e.g., textbooks, scientific research papers, guidelines from medical institutions)?
Scalability	Do the cited references offer further information (e.g., quoted passages, full original text)?

**Table 4:** Inverse-weighted mean ranks of the quality of responses.

Items	Thyro-GenAI	ChatGPT	Perplexity	Claude
A. Factuality	2.9 ± 0.3	2.6 ± 0.2	2.7 ± 0.3	1.9 ± 0.3
A1	2.9 ± 0.4	2.7 ± 0.3	2.7 ± 0.4	1.8 ± 0.4
A2	2.8 ± 0.4	2.4 ± 0.3	2.8 ± 0.4	2.0 ± 0.5
B. Completeness	2.9 ± 0.2	2.3 ± 0.2	2.7 ± 0.2	2.1 ± 0.3
B1	2.9 ± 0.4	2.5 ± 0.3	2.8 ± 0.4	1.8 ± 0.4
B2	2.8 ± 0.3	2.5 ± 0.4	2.7 ± 0.4	2.0 ± 0.5
B3	3.0 ± 0.4	1.9 ± 0.4	2.6 ± 0.4	2.4 ± 0.4
C. Safety	3.1 ± 0.4	2.3 ± 0.3	2.8 ± 0.4	1.7 ± 0.4
C1	3.1 ± 0.4	2.3 ± 0.3	2.8 ± 0.4	1.7 ± 0.4
D. ClinicalApplicability	3.2 ± 0.3	2.1 ± 0.2	2.9 ± 0.3	1.9 ± 0.3
D1	3.2 ± 0.4	2.1 ± 0.4	2.9 ± 0.4	2.0 ± 0.4
D2	3.1 ± 0.4	2.1 ± 0.3	2.9 ± 0.4	1.9 ± 0.4
E. Preference	3.1 ± 0.4	2.2 ± 0.4	2.9 ± 0.4	1.8 ± 0.4
Overall	3.0 ± 0.1	2.3 ± 0.1	2.8 ± 0.1	1.9 ± 0.1

Each value represents the inverse-weighted mean rank ± standard error (SE) for ranks across three evaluators.

**Table 5:** Inverse-weighted mean ranks of the quality of references used.

Items	Thyro-GenAI	ChatGPT	Perplexity	Claude
Hallucination	3.4 ± 0.2	1.9 ± 0.2	3.4 ± 0.3	1.6 ± 0.3
Relevance	2.7 ± 0.5	2.6 ± 0.3	3.1 ± 0.4	1.9 ± 0.3
Quality	3.2 ± 0.4	2.1 ± 0.2	3.0 ± 0.4	2.0 ± 0.4
Scalability	3.2 ± 0.4	2.4 ± 0.2	3.3 ± 0.3	1.5 ± 0.3
Overall	3.1 ± 0.2	2.3 ± 0.1	3.2 ± 0.2	1.8 ± 0.2

Each value represents the inverse-weighted mean rank ± standard error (SE) for ranks across three evaluators.

## Data Availability

The original contributions presented in this study are included in the article/Supplementary Materials. Further inquiries can be directed to the corresponding authors.

## Supplementary Materials

The following supporting information can be downloaded at: https://www.mdpi.com/article/10.3390/jcm14072450/s1; Supplementary Material S1: List of materials used for Thyro-GenAI database; Supplementary Material S2: Generated responses of each chatbot per question; Supplementary Material S3: Free text evaluation.
