# Supplementary material for: Thyro-GenAI: A Chatbot Using Retrieval-Augmented Generative Models for Personalized Thyroid Disease Management
Source: J Clin Med. 2025 Apr 3;14(7):2450. doi: 10.3390/jcm14072450 (PMC11989359; doi:10.3390/jcm14072450)
Supplement: Supplementary file 1 [file jcm-14-02450-s001.zip › Supplementary Material S1.pdf]

Supplemental Data S1. List of materials used for Thyro-GenAI database

Type	Source/Publisher	File format	Title
Textbook	Elsevier	pdf	Surgery of the Thyroid and Parathyroid Glands: Expert Consult Premium Edition - Enhanced Online Features and Print
Textbook	Elsevier	pdf	Sabiston Textbook of Surgery: The Biological Basis of Modern Surgical Practice
Other	Oxford University Hospitals	pdf	Radioactive Iodine Treatment for Hyperthyroidism: Information for Patients
Other	Cardinal Health	pdf	Guidelines for patients receiving radioiodine treatment
Other	Leeds Cancer Centre	pdf	Radioactive Iodine Treatment (Radioiodine): Information for Patients
Other	Memorial Sloan Kettering Cancer Center	pdf	Low Iodine Diet: Memorial Sloan Kettering Cancer Center
Other	American Thyroid Association	pdf	Radioactive Iodine: American Thyroid Association
Other	Memorial Sloan Kettering Cancer Center	pdf	Radioactive Iodine Therapy for Thyroid Cancer: Outpatient Treatment
Guidelines	American Thyroid Association (ATA)	pdf	Consensus Statement on the Terminology and Classification of Central Neck Dissection for Thyroid Cancer
Guidelines	American Thyroid Association (ATA)	pdf	American Thyroid Association Consensus Review and Statement Regarding the Anatomy, Terminology,<br>and Rationale for Lateral Neck Dissection in Differentiated Thyroid Cancer
Guidelines	American Thyroid Association (ATA)	pdf	American Thyroid Association Statement on Outpatient Thyroidectomy
Guidelines	American Thyroid	pdf	American Thyroid Association Statement on Optimal Surgical Management of Goiter

	Association (ATA)		
Guidelines	American Thyroid Association (ATA)	pdf	American Thyroid Association Statement on Preoperative Imaging for Thyroid Cancer Surgery
Guidelines	American Thyroid Association (ATA)	pdf	Management of Recurrent/Persistent Nodal Disease in Patients with Differentiated Thyroid Cancer: A Critical Review of the Risks and Benefits of Surgical Intervention Versus Active Surveillance
Guidelines	American Thyroid Association (ATA)	pdf	American Thyroid Association Statement on Surgical Application of Molecular Profiling for Thyroid Nodules: Current Impact on Perioperative Decision Making
Guidelines	American Thyroid Association (ATA)	pdf	American Thyroid Association Statement on Postoperative Hypoparathyroidism: Diagnosis, Prevention, and Management in Adults
Guidelines	American Thyroid Association (ATA)	pdf	Radiation Safety in the Treatment of Patients with Thyroid Diseases by Radioiodine 131I: Practice Recommendations of the American Thyroid Association
Guidelines	American Thyroid Association (ATA)	pdf	Clinical and Professional Ethics Guidelines for the Practice of Thyroidology
Guidelines	American Thyroid Association (ATA)	pdf	Guidelines for the Treatment of Hypothyroidism Prepared by the American Thyroid Association Task Force on Thyroid Hormone Replacement
Guidelines	American Thyroid Association (ATA)	pdf	Revised American Thyroid Association Guidelines for the Management of Medullary Thyroid Carcinoma
Guidelines	American Thyroid Association (ATA)	pdf	Management Guidelines for Children with Thyroid Nodules and Differentiated Thyroid Cancer
Guidelines	American Thyroid Association (ATA)	pdf	2016 American Thyroid Association Guidelines for Diagnosis and Management of Hyperthyroidism and Other Causes of Thyrotoxicosis
Guidelines	American Thyroid Association (ATA)	pdf	2017 Guidelines of the American Thyroid Association for the Diagnosis and Management of Thyroid Disease During Pregnancy and the Postpartum
Guidelines	American Thyroid Association (ATA)	pdf	Management of Thyroid Eye Disease: A Consensus Statement by the American Thyroid Association and the European Thyroid Association
Guidelines	American Thyroid	pdf	2021 American Thyroid Association Guidelines for Management of Patients with

	Association (ATA)		Anaplastic Thyroid Cancer
Guidelines	European Thyroid Association (ETA)	pdf	2014 European Thyroid Association Guidelines for the Management of Subclinical Hypothyroidism in Pregnancy and in Children
Guidelines	European Thyroid Association (ETA)	pdf	2018 European Thyroid Association (ETA) Guidelines for the Management of Amiodarone-Associated Thyroid Dysfunction
Guidelines	European Thyroid Association (ETA)	pdf	2018 European Thyroid Association (ETA) Guidelines on the Diagnosis and Management of Central Hypothyroidism
Guidelines	European Thyroid Association (ETA)	pdf	2018 European Thyroid Association Guideline for the Management of Graves' Hyperthyroidism
Guidelines	European Thyroid Association (ETA)	pdf	2019 European Thyroid Association Guidelines for the Treatment and Follow-Up of Advanced Radioiodine-Refractory Thyroid Cancer
Guidelines	European Thyroid Association (ETA)	pdf	2019 European Thyroid Association Guidelines on the Management of Thyroid Dysfunction following Immune Reconstitution Therapy
Guidelines	European Thyroid Association (ETA)	pdf	2020 European Thyroid Association Clinical Practice Guideline for the Use of Image-Guided Ablation in Benign Thyroid Nodules
Guidelines	European Thyroid Association (ETA)	pdf	2021 European Thyroid Association Guideline on Thyroid Disorders prior to and during Assisted Reproduction
Guidelines	European Thyroid Association (ETA)	pdf	2021 European Thyroid Association Guidelines for the Management of Iodine-Based Contrast Media-Induced Thyroid Dysfunction
Guidelines	European Thyroid Association (ETA)	pdf	2022 ETA Consensus Statement: What are the indications for post-surgical radioiodine therapy in differentiated thyroid cancer?
Guidelines	European Thyroid Association (ETA)	pdf	2022 European Thyroid Association Guideline for the management of pediatric Graves' disease
Guidelines	European Thyroid Association (ETA)	pdf	2022 European Thyroid Association Guidelines for the management of pediatric thyroid nodules and differentiated thyroid carcinoma
Guidelines	European Thyroid Association (ETA)	pdf	2023 European Thyroid Association Clinical Practice Guidelines for thyroid nodule management

Guidelines	European Thyroid Association (ETA)	pdf	European Thyroid Association and Cardiovascular and Interventional Radiological Society of Europe 2021 Clinical Practice Guideline for the Use of Minimally Invasive Treatments in Malignant Thyroid Lesions
Guidelines	European Thyroid Association (ETA)	pdf	European Thyroid Association Guidelines for Ultrasound Malignancy Risk Stratification of Thyroid Nodules in Adults: The EU-TIRADS
Guidelines	European Thyroid Association (ETA)	pdf	European Thyroid Association Guidelines regarding Thyroid Nodule Molecular Fine-Needle Aspiration Cytology Diagnostics
Guidelines	European Thyroid Association (ETA)	pdf	Evidence-Based Use of Levothyroxine/Liothyronine Combinations in Treating Hypothyroidism: A Consensus Document
Guidelines	European Thyroid Association (ETA)	pdf	The 2015 European Thyroid Association Guidelines on Diagnosis and Treatment of Endogenous Subclinical Hyperthyroidism
Guidelines	European Thyroid Association (ETA)	pdf	The 2016 European Thyroid Association/European Group on Graves' Orbitopathy Guidelines for the Management of Graves' Orbitopathy
Guidelines	Korean Thyroid Association (KTA)	pdf	2021 Korean Thyroid Imaging Reporting and Data System and Imaging-Based Management of Thyroid Nodules: Korean Society of Thyroid Radiology Consensus Statement and Recommendations
Guidelines	Korean Thyroid Association (KTA)	pdf	Diagnosis and Management of Thyroid Disease during Pregnancy and Postpartum: 2023 Revised Korean Thyroid Association Guidelines
Guidelines	Korean Thyroid Association (KTA)	pdf	Evaluation and Management of Bone Health in Patients with Thyroid Diseases: A Position Statement of the Korean Thyroid Association
Guidelines	Korean Thyroid Association (KTA)	pdf	Management of Subclinical Hypothyroidism: A Focus on Proven Health Effects in the 2023 Korean Thyroid Association Guidelines
Guidelines	Korean Thyroid Association (KTA)	pdf	The Revised 2016 Korean Thyroid Association Guidelines for Thyroid Nodules and Cancers: Differences from the 2015 American Thyroid Association Guidelines
Guidelines	Korean Society of Radiology	pdf	2017 Thyroid Radiofrequency Ablation Guideline: Korean Society of Thyroid Radiology
Guidelines	Korean Society of	pdf	2020 Imaging Guidelines for Thyroid Nodules and Differentiated Thyroid Cancer:

	Radiology		Korean Society of Thyroid Radiology
Guidelines	Korean Society of Radiology	pdf	Clinical applications of Doppler ultrasonography for thyroid disease: consensus statement by the Korean Society of Thyroid Radiology
Guidelines	Korean Society of Radiology	pdf	Feasibility of Adjustable Electrodes for Radiofrequency Ablation of Benign Thyroid Nodules
Guidelines	Korean Society of Radiology	pdf	Quality of Life in Patients Treated with Percutaneous Laser Ablation for Non-Functioning Benign Thyroid Nodules: A Prospective Single-Center Study
Guidelines	Korean Society of Radiology	pdf	Radiofrequency versus Ethanol Ablation for Treating Predominantly Cystic Thyroid Nodules: A Randomized Clinical Trial
Guidelines	Korean Society of Radiology	pdf	Unresolved Clinical Issues in Thermal Ablation of Benign Thyroid Nodules: Regrowth at Long-Term Follow-Up
Guidelines	Korean Society of Radiology	pdf	US-Guided Radiofrequency Ablation for Low-Risk Papillary Thyroid Microcarcinoma: Efficacy and Safety in a Large Population
Guidelines	Japan Thyroid Association and Japan Endocrine Society	pdf	2016 Guidelines for the management of thyroid storm from The Japan Thyroid Association and Japan Endocrine Society (First edition)
Guidelines	Japan Associations of Endocrine Surgeons	pdf	The revised clinical practice guidelines on the management of thyroid tumors by the Japan Associations of Endocrine Surgeons: Core questions and recommendations for treatments of thyroid cancer
Guidelines	Springer	pdf	Treatment of thyroid tumor Japanese clinical guidelines
