# Supplementary material for: Thyro-GenAI: A Chatbot Using Retrieval-Augmented Generative Models for Personalized Thyroid Disease Management
Source: J Clin Med. 2025 Apr 3;14(7):2450. doi: 10.3390/jcm14072450 (PMC11989359; doi:10.3390/jcm14072450)
Supplement: Supplementary file 1 [file jcm-14-02450-s001.zip › Supplementary Material S2.pdf]

Supplemental Data S2. Generated responses of each chatbot per question

<table>
<tr><td colspan="2">Question 1</td></tr>
<tr><td>Thyro-GenAI</td><td>To provide a comprehensive and well-structured response, let's break down the management of a 34-year-old pregnant woman with elevated TSH levels, a 1 cm thyroid nodule, and symptoms of fatigue during the first trimester:
Step 1: Initial Assessment
1. Complete History and Clinical Examination:
o Gather detailed information about the patient's medical history, including any previous thyroid issues, family history of thyroid disease, and other relevant health conditions.
o Conduct a thorough clinical examination to assess the thyroid gland and any other related symptoms.
2. Serum TSH Measurement:
o Elevated TSH levels suggest hypothyroidism, which is common during pregnancy due to increased demand for thyroid hormones.
3. Ultrasound of the Neck:
o The ultrasound has already detected a 1 cm thyroid nodule. It's important to evaluate the characteristics of the nodule (e.g., solid or cystic, presence of calcifications) to assess the risk of malignancy.
Step 2: Risk Stratification and Further Testing</td></tr>
</table>

	1. Thyroid Function Tests:<br>o Measure free T4 and T3 levels to confirm hypothyroidism and assess the severity.<br>2. Fine-Needle Aspiration (FNA) Biopsy:<br>o According to the American Thyroid Association guidelines, FNA is generally recommended for nodules ≥1 cm with suspicious features. However, during pregnancy, the decision to perform FNA may depend on the nodule's characteristics and the patient's overall risk profile.<br>Step 3: Management Plan<br>1. Thyroid Hormone Replacement:<br>o If hypothyroidism is confirmed, initiate levothyroxine therapy to normalize TSH levels. This is crucial for both maternal health and fetal development.<br>2. Monitoring and Follow-Up:<br>o Regularly monitor TSH and free T4 levels every 4-6 weeks during pregnancy to ensure adequate thyroid hormone levels.<br>o Adjust levothyroxine dosage as needed based on thyroid function tests.<br>3. Nodule Surveillance:<br>o If the nodule does not have suspicious features, it may be monitored with periodic ultrasound examinations.<br>o If the nodule shows suspicious features or grows

	significantly, consider FNA after the first trimester or postpartum, depending on clinical judgment.<br>Step 4: Addressing Symptoms<br>o Fatigue Management:<br>o Ensure adequate thyroid hormone replacement to alleviate fatigue.<br>o Encourage a balanced diet, regular physical activity, and sufficient rest.<br>Conclusion<br>The management of a pregnant woman with elevated TSH and a thyroid nodule involves a careful balance of monitoring and treatment to ensure the health of both the mother and the developing fetus. Regular follow-up and collaboration with an endocrinologist are recommended to optimize outcomes.
ChatGPT-4	Given the information provided, here's the recommended approach for managing this pregnant patient with elevated TSH, a detected thyroid nodule, and symptoms:<br><br>1. TSH Level and Thyroid Function Management<br>- Elevated TSH (6.7) during the first trimester is above the recommended range. The upper limit for TSH in the first trimester is generally around 2.5-3.0 mIU/L.<br>- This suggests subclinical or overt hypothyroidism, which is especially concerning in pregnancy due to potential impacts on the fetus (e.g.,

	neurodevelopment) and pregnancy outcomes (e.g., miscarriage, preterm birth).<br>- The next step would be to check free T4 levels to assess the extent of thyroid dysfunction:<br>- If free T4 is low: This would confirm overt hypothyroidism, which requires immediate levothyroxine replacement therapy.<br>- If free T4 is normal: This suggests subclinical hypothyroidism, which may still require levothyroxine treatment due to her pregnancy status.<br><br>2. Thyroid Antibody Testing<br>- Test for thyroid peroxidase antibodies (anti-TPO) to evaluate for autoimmune thyroiditis (Hashimoto's disease), which is a common cause of hypothyroidism in pregnancy.<br>- If positive, it increases the likelihood of the patient developing hypothyroidism and may affect the decision to treat even if subclinical.<br><br>3. Management of the Thyroid Nodule<br>- The 1 cm thyroid nodule detected on ultrasound warrants further evaluation given her family history of thyroid nodules.<br>- Thyroid nodules in pregnancy can often be benign, but given her family history, it's essential to rule out malignancy.<br><br>Recommended next steps:<br>- Thyroid ultrasound should be reviewed to assess features of the

	nodule (e.g., solid vs. cystic, presence of calcifications, irregular borders).<br>- Perform a Fine Needle Aspiration (FNA) biopsy if the nodule has suspicious features or if there are risk factors for malignancy (e.g., family history, rapid growth, concerning ultrasound features).<br>- If the ultrasound shows no high-risk features, the biopsy can sometimes be delayed until after delivery, but in the presence of significant concern, an FNA during pregnancy is safe and appropriate.<br><br>4. Symptom Management<br>- The patient reports feeling constantly sleepy, which could be due to hypothyroidism, pregnancy-related fatigue, or both.<br>- If hypothyroidism is confirmed, starting levothyroxine would likely improve her symptoms.<br><br>Summary of Immediate Next Steps:<br>1. Check free T4 and thyroid antibody levels to confirm the diagnosis and severity.<br>2. Initiate levothyroxine therapy if hypothyroidism (subclinical or overt) is confirmed, with a target TSH level below 2.5 mIU/L during the first trimester.<br>3. Review the thyroid nodule ultrasound findings for any suspicious characteristics.<br>4. Consider FNA biopsy of the nodule if warranted by ultrasound features

	or family history.<br><br>Early management of hypothyroidism in pregnancy is critical to optimizing maternal and fetal outcomes. Adjustments to levothyroxine dosing will be required throughout pregnancy, and TSH levels should be monitored every 4-6 weeks.
Perplexity	Based on the information provided, this patient requires prompt evaluation and management due to her elevated TSH and the presence of a thyroid nodule during pregnancy. Let's break down the approach:<br>Thyroid Function Assessment<br>The patient's TSH level of 6.7 mIU/L at 8 weeks gestation is significantly elevated. According to the American Thyroid Association (ATA) guidelines, TSH levels between 2.5-10 mIU/L during the first trimester warrant consideration for treatment. In this case, treatment is strongly recommended.<br>Thyroid Nodule Evaluation<br>The 1 cm thyroid nodule discovered at 14 weeks gestation requires further investigation:<br>1. A fine needle aspiration (FNA) biopsy should be performed, as it is safe during pregnancy and recommended for nodules greater than 1 cm.<br>2. Additional thyroid function tests, including Free T4 and thyroid peroxidase antibodies (TPOAb), should be conducted.<br>Treatment Approach

1. Levothyroxine Therapy: Given the elevated TSH, treatment with levothyroxine should be initiated immediately. The goal is to maintain maternal TSH <2.5 mIU/L during the first trimester and <3.0 mIU/L during the second and third trimesters.
2. Dose Adjustment: Thyroid function tests should be repeated every 4-6 weeks to ensure adequate dosing. Levothyroxine requirements often increase during pregnancy.
3. Iodine Supplementation: Recommend 200 µg/day of potassium iodide supplementation throughout pregnancy.

Follow-up and Monitoring

1. Regular thyroid function tests every 4-6 weeks until delivery.
2. Ultrasound monitoring of the thyroid nodule, with repeat scans every 3-4 weeks or more frequently if poorly controlled.
3. Referral to an endocrinologist for specialized management of thyroid disease during pregnancy.
4. Obstetric care should include growth scans at 28, 32, and 36 weeks due to the risks associated with hypothyroidism.

Patient Education

Explain to the patient that her fatigue may be related to hypothyroidism rather than just pregnancy symptoms. Discuss the importance of adherence to levothyroxine therapy and regular follow-ups.By following these steps, we can ensure proper management of both the patient's hypothyroidism and thyroid nodule during pregnancy, minimizing risks to both mother and fetus.

Claude	Key Information:<br>1. 34-year-old female, 8 weeks pregnant initially<br>2. TSH 6.7 (elevated for pregnancy)<br>3. Family history of thyroid nodules<br>4. 1 cm nodule found on ultrasound at 14 weeks<br>5. Reports fatigue (though could be pregnancy-related)<br><br>Analysis:<br>- During pregnancy, TSH should ideally be <2.5 mIU/L in the first trimester and <3.0 mIU/L in second/third trimesters<br>- Her TSH of 6.7 indicates hypothyroidism requiring treatment<br>- The nodule, while small, requires evaluation given:<br>- Pregnancy status<br>- Family history<br>- Coexisting hypothyroidism<br><br>Next steps should be:<br>1. Immediate initiation of levothyroxine therapy<br>- Critical for fetal development<br>- Target TSH <2.5 mIU/L<br>- Monitor every 4-6 weeks during pregnancy<br><br>2. Fine-needle aspiration (FNA) of the nodule<br>- 1 cm nodule would normally be below the typical threshold for

	FNA<br>- However, given pregnancy and hypothyroidism, FNA is warranted<br>- FNA is safe during pregnancy<br>- Best performed in second trimester

Question 2	
Thyro-GenAI	To determine the recommended next step in managing a 65-year-old female patient with a 0.8 x 0.8 cm thyroid nodule, mild fatigue, a slightly elevated TSH, and a family history of hyperthyroidism, we need to consider several factors based on the context provided and general medical guidelines.<br>Step-by-Step Analysis:<br>1. Patient Profile:<br>o Age: 65 years old<br>o Nodule Size: 0.8 x 0.8 cm<br>o Symptoms: Mild fatigue<br>o TSH Level: Slightly elevated<br>o Family History: Hyperthyroidism<br>o Personal History: No previous thyroid nodules<br>2. Thyroid Nodule Evaluation:<br>o According to the American Thyroid Association (ATA) guidelines, nodules less than 1 cm are generally not biopsied unless there are suspicious ultrasonographic features or a high-risk clinical history.

	- The nodule size (0.8 cm) is below the typical threshold for fine-needle aspiration (FNA) unless there are suspicious features.<br>3. Thyroid Function Tests:<br>- The slightly elevated TSH suggests possible subclinical hypothyroidism, which could be contributing to the patient's fatigue.<br>- Given the family history of hyperthyroidism, it is important to monitor thyroid function closely.<br>4. Recommended Next Steps:<br>- Thyroid Ultrasound: Perform a neck ultrasound to assess the nodule's characteristics. This will help determine if there are any suspicious features that warrant further investigation.<br>- Monitor TSH Levels: Regular monitoring of TSH and other thyroid function tests (such as free T4) to assess thyroid function over time.<br>- Consider Risk Factors: Given the family history of hyperthyroidism, it is important to consider the potential for thyroid dysfunction and monitor accordingly.<br>- Symptom Management: Address the mild fatigue, possibly related to the slightly elevated TSH, by considering lifestyle modifications or discussing potential treatment options with an endocrinologist.

	Conclusion:<br>The primary focus should be on performing a thyroid ultrasound to evaluate the nodule's characteristics and monitoring thyroid function tests to manage the slightly elevated TSH and associated symptoms. If the ultrasound reveals suspicious features, further evaluation with FNA may be considered. Regular follow-up with an endocrinologist is advisable to ensure comprehensive management of the patient's thyroid health.
ChatGPT-4	Given the information provided, the next step should focus on evaluating the thyroid nodule, particularly since the patient has a slightly elevated TSH level. Here's a detailed plan:<br>Recommended Next Step:<br>1. Thyroid Ultrasound<br>• Rationale: An ultrasound is crucial to assess the characteristics of the nodule (size, composition, echogenicity, presence of microcalcifications, margins, vascularity, etc.). This helps determine whether the nodule is likely benign or suspicious for malignancy.<br>• TSH Interpretation: The slightly elevated TSH suggests possible subclinical hypothyroidism, which may or may not be related to the thyroid nodule. However, the priority is to characterize the nodule first.<br>2. Fine Needle Aspiration Biopsy (FNA) - If Indicated by Ultrasound Findings<br>• When to perform FNA: According to guidelines (such as those

	from the American Thyroid Association), a biopsy is typically recommended if:<br>o The nodule is solid and ≥1 cm with suspicious ultrasound features (e.g., irregular margins, microcalcifications, marked hypoechogenicity).<br>o The nodule is ≥1.5-2 cm if it has intermediate-risk features.<br>o The nodule is ≥2 cm if it appears benign on ultrasound.<br>• For a nodule that is 0.8 cm, an FNA would typically only be indicated if there are suspicious ultrasound features or if there is a significant family history of thyroid cancer.<br>3. Thyroid Function Tests<br>• Consider a repeat TSH, along with Free T4 and T3 levels, to get a clearer picture of the thyroid function status, especially if the slightly elevated TSH persists. This will help rule out or confirm hypothyroidism.<br>Additional Considerations<br>• Family History: The patient's family history of hyperthyroidism in her brother and daughter is noted but doesn't directly correlate with an increased risk of thyroid cancer. However, it may be relevant if a thyroid dysfunction is suspected.<br>• Mild Fatigue: The patient's mild fatigue could be related to the slightly elevated TSH, indicating subclinical hypothyroidism. This may need to be managed depending on her thyroid function

	test results.<br>Summary<br>The next immediate step is to perform a thyroid ultrasound to evaluate the characteristics of the nodule. If concerning features are identified, an FNA biopsy would be warranted, even though the nodule is less than 1 cm. Simultaneously, monitoring thyroid function with repeated TSH and additional thyroid hormone levels will help guide further management.
Perplexity	Based on the information provided, here's the recommended approach for this 65-year-old female patient with a newly discovered thyroid nodule:<br>Initial Assessment<br>The patient has a 0.8 x 0.8 cm thyroid nodule discovered during a routine health examination. She is largely asymptomatic, with only mild fatigue reported. Her TSH is slightly elevated, which suggests potential subclinical hypothyroidism.<br>Next Steps<br>1. Thyroid Function Tests: A complete thyroid function panel should be performed, including free T4 and free T3, to confirm the diagnosis of subclinical hypothyroidism<br>2. Thyroid Ultrasound: A dedicated thyroid ultrasound should be performed to fully characterize the nodule, including its size, composition (solid, cystic, or mixed), echogenicity, margins, and any suspicious features<br>3. Risk Stratification: Based on the ultrasound findings, the nodule should be classified according to a standardized risk stratification

system, such as the EU-TIRADS (European Thyroid Imaging and Reporting Data System)

Further Management

Depending on the results of these initial tests, the following steps may be considered:

1. Fine Needle Aspiration (FNA): Given the patient's age (>60 years) and the nodule size (0.8 cm), FNA may not be immediately necessary unless suspicious ultrasound features are present. The decision for FNA should be based on the risk stratification from the ultrasound.
2. Treatment of Subclinical Hypothyroidism: Given the patient's age (>65 years) and slightly elevated TSH, treatment with levothyroxine should be considered. The initial dose should be low (25-50 mcg daily) and gradually increased to avoid cardiac complications
3. Follow-up: If FNA is not performed initially, close follow-up with repeat ultrasound in 6-12 months is recommended to monitor for any changes in nodule size or characteristics
4. Consideration of Family History: While the patient has a family history of hyperthyroidism, her slightly elevated TSH suggests she is not hyperthyroid. However, this family history should be noted for future monitoring
5. Additional Considerations

	• The patient's use of Ginkgo should be noted, as it may interact with thyroid hormone replacement if initiated<br>• Her history of hyperlipidemia may improve with treatment of subclinical hypothyroidism if confirmed<br>• The mild fatigue reported by the patient may be related to subclinical hypothyroidism and should be reassessed after treatment initiation<br><br>In summary, the next steps should include a complete thyroid function panel, a dedicated thyroid ultrasound, and risk stratification of the nodule. Based on these results, decisions regarding FNA, treatment of subclinical hypothyroidism, and follow-up can be made. The patient's age and overall health status should be considered in all treatment decisions.
Claude	Let me think through this case systematically:<br>Key Information:<br>1. 65-year-old female<br>2. 0.8 x 0.8 cm thyroid nodule (< 1 cm)<br>3. Asymptomatic except mild fatigue<br>4. Elevated TSH<br>5. Family history of hyperthyroidism but not nodules<br>6. Current comorbidity: hyperlipidemia<br>7. Personal history of Bell's palsy<br><br>Analysis:

	- The nodule is small (< 1 cm), which typically would not require immediate FNA based on size alone<br>- However, we have some concerning factors:<br>* Patient's age (>60 years is a risk factor)<br>* Elevated TSH (associated with increased risk of malignancy)<br>* The nodule was an incidental finding<br><br>Given these factors, I recommend:<br><br>1. First step: Complete thyroid function panel including:<br>- Free T4<br>- Free T3<br>- Anti-thyroid antibodies (TPO and TG antibodies)<br>- Thyroglobulin level<br><br>2. Thyroid ultrasound if not already done, to:<br>- Characterize nodule features (solid vs. cystic, echogenicity, margins, calcifications)<br>- Evaluate for cervical lymphadenopathy<br>- Check for other nodules<br>- Determine if FNA is needed based on sonographic features<br><br>3. Consider referral to an endocrinologist for:<br>- Management of apparent subclinical hypothyroidism

	- Monitoring of the nodule<br>- Decision-making regarding FNA based on complete evaluation

Question 3	
Thyro-GenAI	To determine the recommended management for a 0.7 cm thyroid nodule that is taller than wide in a 45-year-old male with normal thyroid function tests and no significant radiation exposure or symptoms, we need to consider several factors based on the guidelines and context provided.<br>Step-by-Step Analysis:<br>1. Nodule Size and Characteristics:<br>o The nodule is 0.7 cm, which is relatively small.<br>o It is described as "taller than wide," which is a suspicious feature on ultrasound, often associated with a higher risk of malignancy.<br>2. Patient Profile:<br>o The patient is 45 years old, which does not fall into the highest risk age groups for thyroid cancer (younger than 30 or older than 60).<br>o There is no significant history of radiation exposure, which reduces the risk of malignancy.<br>3. Thyroid Function:<br>o The patient has normal thyroid function tests, indicating that the nodule is not causing hyperthyroidism or hypothyroidism.

4. Guidelines and Recommendations:
    - According to the American Thyroid Association (ATA) guidelines, fine needle aspiration (FNA) is generally recommended for nodules greater than 1 cm if they have suspicious features.
    - For nodules less than 1 cm, especially those with suspicious features, the decision to perform FNA is more nuanced and often depends on additional risk factors and clinical judgment.

Recommended Management:

Given the nodule's size (0.7 cm) and its suspicious ultrasound feature (taller than wide), but considering the absence of other high-risk factors (such as significant radiation exposure or symptoms), the following management approach is suggested:

- Active Surveillance:
    - Regular follow-up with ultrasound monitoring to assess any changes in the nodule's size or characteristics.
    - This approach is often preferred for small nodules with suspicious features but without other high-risk factors.
- Consideration for FNA:
    - If there are any changes in the nodule's characteristics or if the patient develops symptoms, an FNA may be considered to rule out malignancy.
- Patient Education:

	◦ Inform the patient about the potential risks and benefits of active surveillance versus immediate biopsy.<br>◦ Discuss the importance of regular follow-up and monitoring.<br>Visual Summary:<br>◦ Nodule Size: 0.7 cm<br>◦ Ultrasound Feature: Taller than wide (suspicious)<br>◦ Patient Age: 45 years<br>◦ Thyroid Function: Normal<br>◦ Radiation Exposure: None<br>◦ Recommended Action: Active surveillance with regular ultrasound follow-up<br>This approach balances the need for vigilance due to the suspicious feature with the understanding that the nodule is small and the patient has no other high-risk factors.
ChatGPT-4	Given the findings, particularly the fact that the thyroid nodule is taller than wide, this is considered a suspicious ultrasound feature that raises concern for malignancy. Here's a breakdown of the next steps:<br>Key Points:<br>1. Thyroid Nodule Size and Shape:<br>◦ The nodule is 0.7 cm and taller than wide, which is a significant ultrasound finding associated with an increased risk of thyroid cancer.<br>2. Patient's History:

	- No history of radiation exposure.<br>- No symptoms related to the thyroid.<br>- Normal thyroid function tests.<br>- History of hepatitis B and varicose vein treatment does not directly affect thyroid nodule management.<br>Next Step in Management:<br>The recommended next step would be:<br>1. Fine-Needle Aspiration (FNA) Biopsy<br>• For a nodule that is taller than wide, even if it is relatively small (0.7 cm), an FNA biopsy is often indicated due to the suspicious ultrasound characteristics.<br>• Guidelines (e.g., American Thyroid Association) generally recommend FNA for nodules ≥1 cm if they have suspicious features. However, a taller-than-wide shape is considered suspicious enough to warrant an FNA biopsy, even in nodules smaller than 1 cm.<br>2. Additional Considerations:<br>• Thyroid Ultrasound: Ensure that a high-quality ultrasound is performed to evaluate additional suspicious features like microcalcifications, irregular borders, or hypoechogenicity.<br>• Thyroid Function Tests: While they are currently normal, monitoring might be needed if any intervention is planned.<br>Summary:<br>The most appropriate next step is to proceed with an FNA biopsy of the

	thyroid nodule due to its suspicious ultrasound feature (taller-than-wide shape). This will help rule out malignancy and guide further management.
Perplexity	Based on the information provided, the next step in managing this patient's thyroid nodule should be further evaluation with ultrasound-guided fine needle aspiration (FNA). Here's a detailed explanation of the rationale:<br>Nodule Characteristics and Risk Assessment<br>The thyroid nodule in question has several features that warrant further investigation:<br>1. Size: At 0.7 cm, the nodule is close to the 1 cm threshold typically used for recommending FNA. While some guidelines suggest a more conservative approach for nodules <1 cm, the presence of suspicious ultrasound features may lower this threshold.<br>2. Shape: The nodule is described as "taller than wide," which is an important sonographic feature associated with an increased risk of malignancy. This shape has been reported to have an odds ratio of 11.1 for malignancy<br>3. Patient age: At 45 years old, the patient is in an age group where thyroid nodules have a higher likelihood of malignancy compared to younger individuals.<br>Recommendation for FNA<br>Given these factors, particularly the taller-than-wide shape, which is a strong indicator of potential malignancy, the most appropriate next step

	would be to perform an ultrasound-guided FNA<br><br>This procedure is recommended for:<br><br>• Nodules with suspicious sonographic characteristics<br>• Nodules larger than 1-1.5 cm (though exceptions are made for smaller nodules with suspicious features)<br>• Cases where clinical risk factors for malignancy are present<br><br>Additional Considerations<br><br>1. Thyroid Function: The patient's normal thyroid function tests are reassuring but do not rule out malignancy.<br>2. Medical History: While the patient's history of hepatitis B and varicose vein treatment are not directly related to thyroid cancer risk, they should be considered in the overall management plan.<br>3. Occupation: Although the patient works in a hospital, there's no mention of specific radiation exposure, which could have been a risk factor for thyroid cancer.<br><br>Conclusion<br><br>While the nodule is slightly smaller than the typical 1 cm threshold, its taller-than-wide shape is a significant concern. Therefore, proceeding with ultrasound-guided FNA is the most prudent next step to definitively assess the nature of this nodule and guide further management decisions. This approach aligns with current guidelines for thyroid nodule management and provides the best opportunity for early detection and

	treatment if malignancy is present.
Claude	Let me analyze this case step by step:<br>Key Information:<br>1. 45-year-old male<br>2. 0.7cm thyroid nodule<br>o Taller than wide (suspicious ultrasound feature)<br>o Found incidentally<br>3. No radiation exposure history<br>4. Asymptomatic<br>5. Normal TFT<br>6. Relevant medical history:<br>o Hepatitis B carrier<br>o On medication for varicose veins<br>Assessment: While the nodule is small (< 1cm), it has a suspicious sonographic feature (taller-than-wide shape), which increases the risk of malignancy. According to the American Thyroid Association (ATA) guidelines:<br>Next steps should be:<br>1. Primary recommendation:<br>• Ultrasound follow-up of the nodule in 6-12 months<br>• This is because despite the suspicious feature, the nodule is < 1cm, and current guidelines generally don't recommend FNA (Fine Needle Aspiration) for nodules < 1cm unless there are other high-risk features

	2. Additional measures:<br>• Document detailed ultrasound characteristics (echogenicity, composition, margins, calcifications)<br>• Continue regular TFT monitoring<br>• Ensure HBV treatment is optimized as concurrent management<br>• No need to adjust varicose vein medication<br>3. Patient education about:<br>• Warning signs to watch for (rapid growth, hoarseness, difficulty swallowing)<br>• Importance of follow-up adherence

Question 4	
Thyro-GenAI	To determine the recommended management for a 47-year-old male with a 6.8 mm thyroid nodule classified as K-TIRADS 4/5, we need to consider the following factors:<br>1. K-TIRADS Classification:<br>o K-TIRADS 4/5 indicates a high suspicion of malignancy. K-TIRADS 4 is considered "moderate to high suspicion," while K-TIRADS 5 is "high suspicion" for malignancy.<br>2. Nodule Size:<br>o The nodule is 6.8 mm, which is relatively small. According to the guidelines, fine needle aspiration (FNA) is generally recommended for nodules larger than 1 cm, even with highly suspicious features. However, for

	nodules smaller than 1 cm, the decision to perform FNA is more conservative and often based on other risk factors.<br>3. Patient's Medical History:<br>o The patient has no significant family or surgical history related to thyroid cancer, which reduces the risk factors.<br>o The patient is on medication for hypertension and hyperlipidemia, which are not directly related to thyroid nodule management but should be considered in the overall health assessment.<br>4. Guidelines and Recommendations:<br>o According to the latest American Thyroid Association (ATA) guidelines and the Korean Thyroid Association, for nodules less than 1 cm with high suspicion features (K-TIRADS 4/5), active surveillance with periodic ultrasound monitoring is often recommended rather than immediate FNA or surgery, unless there are other risk factors or symptoms.<br>Recommended Management:<br>o Active Surveillance:<br>o Regular follow-up with ultrasound every 6-12 months to monitor for any changes in size or characteristics of the nodule.<br>o Consideration for FNA:<br>o If the nodule shows significant growth or changes in

	ultrasound features during follow-up, an FNA may be considered to rule out malignancy.<br>o Patient Counseling:<br>o Discuss the potential risks and benefits of active surveillance versus immediate intervention with the patient, considering his overall health and preferences.<br>o Management of Comorbidities:<br>o Continue managing hypertension and hyperlipidemia as per the current treatment plan, ensuring these conditions are well-controlled.<br>This approach balances the need for vigilance due to the high suspicion classification with the conservative management typically recommended for small nodules.
ChatGPT-4	Given the information provided, the next step in managing a thyroid nodule that is 6.8 mm and classified as K-TIRADS 4/5 is to consider the following options based on current guidelines:<br>1. Fine-Needle Aspiration (FNA) Biopsy<br>• K-TIRADS 4 and K-TIRADS 5 nodules are considered moderate to high suspicion for malignancy. However, the size threshold for FNA in such high-risk nodules is typically >10 mm.<br>• Given that the nodule in this case is only 6.8 mm, it falls below the threshold where FNA biopsy is usually recommended.<br>2. Active Surveillance<br>• For nodules less than 10 mm that are K-TIRADS 4 or 5, active

	surveillance with periodic ultrasound follow-up is generally recommended rather than immediate FNA.<br>• Typically, the follow-up ultrasound is done in 6-12 months to monitor for any changes in size or characteristics that might warrant a biopsy later.<br>3. Consideration of Patient Factors<br>• In a patient with no significant family history of thyroid cancer, no rapid growth of the nodule, and no suspicious lymphadenopathy, conservative management with regular ultrasound follow-up is usually appropriate.<br>• If the patient has specific risk factors (e.g., family history of thyroid cancer, radiation exposure) or exhibits worrisome symptoms (e.g., voice changes, dysphagia), FNA may be considered even if the nodule is smaller than 10 mm.<br>Conclusion<br>The most appropriate next step in this patient's management would be:<br>• Active surveillance with a follow-up ultrasound in 6-12 months.<br>• FNA biopsy is not immediately indicated given the nodule's size (6.8 mm) despite its high K-TIRADS classification.<br>This approach balances the risk of over-diagnosis and overtreatment while ensuring close monitoring of a potentially suspicious nodule.
Perplexity	Based on the information provided, the next step in managing this patient's thyroid nodule should be carefully considered, taking into account the K-TIRADS classification, nodule size, and patient factors.

	Let's break down the key points and recommendations:<br>Nodule Characteristics and Classification<br>The thyroid nodule in question is 6.8 mm in size and classified as K-TIRADS 4/5. This classification indicates an intermediate to high suspicion for malignancy:<br>• K-TIRADS 4 nodules present an intermediate malignancy risk of 10-40%.<br>• K-TIRADS 5 nodules are considered high suspicion for malignancy, with a risk of about 73.4%.<br>Biopsy Recommendations<br>According to the 2021 Korean Thyroid Imaging Reporting and Data System (K-TIRADS) guidelines:<br>• For K-TIRADS 4 nodules, biopsy is recommended for nodules measuring 1-1.5.<br>• For K-TIRADS 5 nodules, biopsy is recommended for nodules ≥1 cm, with some cases considering biopsy for nodules >0.5 .<br><br>Next Steps<br>Given that the nodule is 6.8 mm, which is below the typical size threshold for biopsy in both K-TIRADS 4 and 5 categories, the next steps should be:<br>1. Close follow-up: Since the nodule is smaller than the recommended size for immediate biopsy, close monitoring with regular ultrasound examinations is advisable. This approach

	allows for tracking any changes in size or characteristics over time.<br>2. Repeat ultrasound: Schedule a follow-up ultrasound in 6-12 months to reassess the nodule's size and features. If there's significant growth or changes in suspicious characteristics, earlier intervention may be warranted.<br>3. Consider patient factors: While the patient has no significant family history or prior thyroid issues, the presence of hypertension and hyperlipidemia should be noted as part of the overall health assessment.<br>4. Patient education: Inform the patient about the nature of the nodule, the reasons for monitoring rather than immediate biopsy, and signs or symptoms to watch for (e.g., difficulty swallowing, voice changes, neck discomfort).<br>5. Interdisciplinary consultation: Consider discussing the case with an endocrinologist or thyroid specialist to ensure the most appropriate management plan, especially given the borderline K-TIRADS classification (4/5).<br>It's important to note that while K-TIRADS provides valuable guidance, clinical judgment should always be exercised. If there are any additional concerning features or if the patient expresses strong preference for more aggressive management, fine-needle aspiration (FNA) biopsy could be considered despite the small size, particularly if it's classified as K-TIRADS 5.

	In conclusion, the next step should be close follow-up with a repeat ultrasound in 6-12 months, unless additional risk factors or patient preferences necessitate earlier intervention. This approach balances the need for vigilance with avoiding unnecessary invasive procedures for a small nodule.
Claude	Let me help analyze this case step by step:<br>Key Information:<br>1. 47-year-old male<br>2. Thyroid nodule discovered incidentally<br>3. Nodule size: 6.8 mm<br>4. K-TIRADS 4/5 (high suspicion for malignancy)<br>5. No previous neck masses<br>6. Only medications are for HTN and hyperlipidemia<br>7. No relevant family/surgical history<br>Analysis:<br>• K-TIRADS 4/5 indicates suspicious sonographic features that warrant further evaluation<br>• However, the nodule size (6.8 mm) is less than 1 cm, which is an important threshold in management decisions<br>• The patient has no high-risk history factors that would lower the threshold for intervention<br>Recommendation: The next appropriate step would be active surveillance with a follow-up ultrasound in 6-12 months, rather than immediate fine-

	needle aspiration (FNA). This recommendation is based on:<br>1. While K-TIRADS 4/5 suggests high suspicion, the small size (<1 cm) makes the risk of clinically significant cancer low<br>2. Current guidelines (American Thyroid Association) generally recommend FNA for highly suspicious nodules only when they are ≥1 cm<br>3. The patient has no concerning risk factors that would warrant more aggressive management

Question 5	
Thyro-GenAI	To determine the recommended management for a 56-year-old woman with a thyroid nodule that has increased in size from 0.8 cm to 1.2 cm over four years and is classified as K-TIRADS 3, we need to consider several factors based on the provided context and general guidelines for thyroid nodule management.<br>Step-by-Step Analysis:<br>1. Nodule Classification and Size:<br>o The nodule is classified as K-TIRADS 3, which indicates a low to intermediate suspicion of malignancy.<br>o The size of the nodule has increased from 0.8 cm to 1.2 cm over four years.<br>2. K-TIRADS Guidelines:<br>o According to the 2021 K-TIRADS guidelines, biopsy is recommended for K-TIRADS 3 nodules that are greater

	than 2 cm in size. Since the nodule is 1.2 cm, it does not meet the size criteria for an immediate biopsy based on K-TIRADS 3 classification.<br>3. Growth Over Time:<br>o The nodule has shown growth over the years, which can be a factor to consider for further evaluation. However, the increase in size is relatively small over a four-year period.<br>4. Patient Factors:<br>o The patient is 56 years old with no medication use or family history of thyroid disease, which does not add additional risk factors for malignancy.<br>5. Recommended Management:<br>o Given the nodule's classification, size, and growth pattern, the recommended management would likely involve continued surveillance rather than immediate intervention.<br>o Regular follow-up with ultrasound imaging to monitor any further changes in size or characteristics of the nodule is advisable.<br>o If the nodule continues to grow or if there are changes in its ultrasound features that increase suspicion, a fine-needle aspiration (FNA) biopsy may be considered in the future.<br>Conclusion:<br>For this patient, the current management strategy would be continued

	surveillance with regular ultrasound follow-ups. This approach allows for monitoring any significant changes that might warrant further investigation, such as an FNA biopsy, especially if the nodule grows beyond the 2 cm threshold or exhibits suspicious features. Regular follow-up intervals can be determined by the healthcare provider based on clinical judgment and patient-specific factors.
ChatGPT-4	Given the increase in size of the thyroid nodule from 0.8 cm to 1.2 cm over the span of 4 years, the next appropriate step would be to perform a fine-needle aspiration (FNA) biopsy. Here's the rationale:<br>Clinical Considerations:<br>1. K-TIRADS 3 (Intermediate Risk) Classification:<br>o K-TIRADS 3 nodules have an intermediate suspicion for malignancy (estimated risk: 5-15%).<br>o According to K-TIRADS guidelines, FNA is generally recommended if:<br>▪ The nodule is ≥1.0 cm in size and shows growth or suspicious features.<br>2. Nodule Growth:<br>o An increase in size from 0.8 cm to 1.2 cm represents a significant change.<br>o Growth is defined as a ≥20% increase in at least two dimensions with a minimal increase of 2 mm in solid components or an increase in nodule volume.<br>3. Patient History and Risk Factors:

	◦ Although there is no family history of thyroid cancer or associated risk factors, the increase in size warrants further investigation to rule out malignancy.<br>Recommended Next Step:<br>Fine-Needle Aspiration (FNA) Biopsy:<br>• This is the most appropriate step to evaluate the nature of the nodule and determine if it is benign or malignant.<br>• FNA is indicated for K-TIRADS 3 nodules ≥1.0 cm, especially if there is documented growth over time.<br>Additional Recommendations:<br>• If FNA results are indeterminate, molecular testing or repeat ultrasound may be considered.<br>• If FNA results are benign, continued ultrasound surveillance every 1-2 years may be appropriate, depending on clinical context.<br>Given the patient's history of interval growth, it is crucial to proceed with FNA to obtain a cytological diagnosis.
Perplexity	Based on the information provided and the current guidelines, here's the recommended approach for this patient:<br>Assessment<br>The patient has a thyroid nodule that was initially classified as K-TIRADS 3 (low suspicion) with a size of 0.8 cm. After 4 years, the nodule has grown to 1.2 cm, showing a significant increase in size.<br>Recommended Next Steps

	1. Ultrasound-guided Fine Needle Aspiration Biopsy (FNAB)<br>The next step for this patient should be to perform an ultrasound-guided fine needle aspiration biopsy (FNAB) of the thyroid nodule. This recommendation is based on several factors:<br>• The nodule has increased in size from 0.8 cm to 1.2 cm over 4 years, which represents a significant growth (greater than 2 mm in at least two dimensions and a diameter increase of more than 20%).<br>• The current size of 1.2 cm meets the criteria for biopsy in K-TIRADS 3 nodules according to the modified K-TIRADS guidelines.<br><br>Detailed Ultrasound Examination<br>Before proceeding with the biopsy, a detailed ultrasound examination should be performed to:<br>• Reassess the K-TIRADS classification of the nodule, as its features may have changed with growth.<br>• Evaluate for any new suspicious ultrasound features that may have developed.<br>Follow-up Plan<br>After the FNAB results are obtained:<br>• If the biopsy confirms benignity, follow-up ultrasound should be performed within 12-24 months.<br>• If the biopsy is inconclusive or shows atypical cells, further

	management will depend on the specific cytological findings and clinical context.<br>Long-term Management<br>Regardless of the biopsy results, this patient will require ongoing surveillance:<br>• For K-TIRADS 3 nodules, ultrasound follow-up should be performed at 1, 3, and 5 years.<br>• If there are no significant changes in nodule size or characteristics after 5 years of stable follow-up, the interval between ultrasounds may be extended.<br>By following these steps, we can ensure appropriate management of the patient's thyroid nodule, balancing the need for diagnostic accuracy with the goal of minimizing unnecessary procedures.
Claude	Let me think through this case step by step:<br>1. Key Patient Information:<br>• 56-year-old woman<br>• Left thyroid nodule: initially 0.8 cm, now 1.2 cm (50% increase in 4 years)<br>• K-TIRADS 3 (intermediate suspicion)<br>• No medications or family history of thyroid disease<br>• Lost to follow-up for 4 years<br>2. Risk Assessment:<br>• K-TIRADS 3 indicates intermediate suspicion for malignancy (3-15%)

	• The nodule has shown significant growth (>50% increase in volume)<br>• Growth of nodule during surveillance is a concerning feature<br>3. K-TIRADS 3 Management Guidelines:<br>• Nodules ≥1.0 cm typically warrant FNA<br>• Current size (1.2 cm) exceeds this threshold<br>• Growth during surveillance adds to suspicion<br>4. Additional Concerning Factors:<br>• Lost to follow-up period means delayed surveillance<br>• Patient is in an age group where thyroid cancer is possible<br>Next Step: The most appropriate next step would be to perform a Fine Needle Aspiration (FNA) biopsy of the nodule. This is indicated because:<br>• The nodule is now >1 cm<br>• There is documented growth<br>• The K-TIRADS 3 classification indicates intermediate suspicion<br>• The long gap in surveillance necessitates definitive evaluation<br>In addition to FNA, current thyroid function tests (TSH, free T4) should be obtained if not recently done.

Question 6	
Thyro-GenAI	When considering obesity treatment options for a 48-year-old woman with an 8 mm thyroid nodule and a family history of papillary thyroid cancer, it's important to take into account her specific medical history and contraindications. Since Saxenda (liraglutide) is contraindicated due to

	her family history, alternative approaches should be explored. Here's a detailed breakdown of potential options:<br>1. Lifestyle Modifications<br>o Dietary Changes: Implementing a balanced, calorie-controlled diet rich in fruits, vegetables, lean proteins, and whole grains. Consulting with a registered dietitian can provide personalized guidance.<br>o Physical Activity: Encouraging regular physical activity, such as brisk walking, swimming, or cycling, aiming for at least 150 minutes of moderate-intensity exercise per week.<br>2. Behavioral Therapy<br>o Counseling: Engaging in behavioral therapy to address eating habits, emotional eating, and motivation. Cognitive-behavioral therapy (CBT) can be particularly effective.<br>o Support Groups: Joining weight loss support groups or programs like Weight Watchers for community support and accountability.<br>3. Pharmacotherapy<br>o Alternative Medications: Considering other weight loss medications that do not have the same contraindications as Saxenda. Options may include orlistat, which works by reducing fat absorption, or other medications as deemed appropriate by her healthcare provider.<br>4. Surgical Options<br>o Bariatric Surgery: If lifestyle modifications and pharmacotherapy

	are insufficient, and if she meets the criteria, bariatric surgery could be considered. Options include gastric bypass, sleeve gastrectomy, or adjustable gastric banding. A thorough evaluation by a bariatric surgeon is necessary to assess suitability.<br>5. Monitoring and Management of Thyroid Nodule<br>o Regular Follow-up: Given the family history of papillary thyroid cancer, regular monitoring of the thyroid nodule through ultrasound and possibly fine-needle aspiration (FNA) biopsy may be warranted to assess any changes in size or characteristics.<br>o Endocrinologist Consultation: Consulting with an endocrinologist for comprehensive management of thyroid health and to evaluate any potential impact on weight management strategies.<br>6. Holistic and Complementary Approaches<br>o Mindfulness and Stress Reduction: Techniques such as yoga, meditation, or tai chi can help manage stress, which may contribute to weight gain.<br>o Nutritional Supplements: While not a primary treatment, certain supplements like fiber or protein shakes can support dietary efforts, but should be used under medical guidance.<br>Conclusion<br>It's crucial to have a multidisciplinary approach involving healthcare providers, including a primary care physician, endocrinologist, dietitian, and possibly a bariatric specialist, to tailor a safe and effective weight management plan. Regular monitoring and adjustments based on her

	progress and any changes in her thyroid condition are essential.
ChatGPT-4	Given her history, including a family history of papillary thyroid cancer and the presence of a thyroid nodule, it’s wise to avoid medications like Saxenda (liraglutide) due to its contraindications related to thyroid cancer risk. However, there are several other options available for obesity management that could be considered. Here's a detailed overview:<br>1. Lifestyle Modifications<br>• Dietary changes: A structured, calorie-restricted diet (e.g., low-carb, Mediterranean, or DASH diet).<br>• Physical activity: Aiming for at least 150 minutes of moderate-intensity exercise per week.<br>• Behavioral therapy: Involving structured weight management programs or counseling.<br>2. Pharmacotherapy<br>Since Saxenda is contraindicated, other weight-loss medications that do not have the same contraindications may be suitable. Some alternatives include:<br>• Orlistat (Xenical, Alli): Works by reducing fat absorption in the intestines. It does not have thyroid-related contraindications, but it may cause gastrointestinal side effects.<br>• Phentermine/Topiramate (Qsymia): A combination medication that suppresses appetite and increases energy expenditure. It should be used cautiously, especially if the patient has hypertension, but may be an option if her blood pressure is well-

	controlled.<br>• Naltrexone/Bupropion (Contrave): This combination helps reduce appetite and control cravings. It is generally safe regarding thyroid conditions but should be avoided if the patient has uncontrolled hypertension.<br>• Metformin: While not officially approved for weight loss, it can lead to modest weight loss, especially in patients with insulin resistance or pre-diabetes.<br>• Semaglutide (Wegovy): Another GLP-1 agonist like Saxenda but with a different molecular structure. Although it’s important to verify its safety in the context of thyroid cancer risk, the contraindications might be less strict than those for liraglutide.<br>3. Non-Pharmacological Interventions<br>• Endoscopic procedures: These are minimally invasive options, such as endoscopic sleeve gastroplasty, which may be appropriate for individuals who are not candidates for surgery.<br>• Intragastric balloon: A balloon is temporarily placed in the stomach to promote a feeling of fullness.<br>4. Surgical Options<br>For patients with a higher BMI or when other interventions have failed:<br>• Bariatric surgery (e.g., sleeve gastrectomy, gastric bypass) is a highly effective option for significant weight loss and can improve comorbidities like hypertension and diabetes.<br>Considerations Given Her Medical History

	• Careful monitoring of her thyroid status is essential, especially with her history of hypothyroidism and thyroid nodule.<br>• It would be beneficial to perform a fine-needle aspiration biopsy (FNA) of the thyroid nodule, given its size (8 mm) and her significant family history of papillary thyroid cancer. This can help determine if further evaluation or treatment of the nodule is necessary before initiating obesity therapy.<br>In summary, lifestyle changes, medications like Orlistat, Qsymia, or Contrave, and possibly surgical interventions should be explored while avoiding medications like Saxenda that may exacerbate thyroid cancer risk.
Perplexity	Given the patient's medical history and contraindication for Saxenda, there are several alternative options to consider for her obesity treatment:<br>Alternative Medications<br>1. Wegovy (semaglutide): This is another GLP-1 receptor agonist like Saxenda, but it's administered once weekly instead of daily. It has shown significant efficacy for weight loss in clinical trials. However, like Saxenda, it's contraindicated in patients with a personal or family history of medullary thyroid carcinoma, so it may not be suitable given the patient's family history of thyroid cancer.<br>2. Xenical (orlistat): This medication works by inhibiting fat absorption in the digestive system. It's a different approach from GLP-1 agonists and doesn't have the same contraindications

related to thyroid cancer.

3. Qsymia (phentermine-topiramate): This combination medication can be effective for weight loss. It doesn't have specific contraindications related to thyroid cancer.
4. Contrave (naltrexone-bupropion): Another combination medication that can aid in weight loss without thyroid cancer-related contraindications.

Non-Pharmacological Approaches

1. Dietary modifications: A balanced, calorie-restricted diet tailored to the patient's needs can be effective for weight loss.
2. Increased physical activity: Regular exercise, combining both aerobic and anaerobic activities, can aid in weight loss and overall health improvement.
3. Behavioral therapy: This can help the patient develop healthier eating habits and maintain long-term lifestyle changes.

Considerations

- Given the patient's thyroid nodule and family history of thyroid cancer, close monitoring of her thyroid function and the nodule is crucial.
- The patient is already on levothyroxine for hypothyroidism. Ensuring optimal thyroid hormone replacement is important, as hypothyroidism can contribute to weight gain.
- Any weight loss intervention should be implemented alongside continued management of her hypertension and hypothyroidism.

	- A comprehensive approach combining dietary changes, increased physical activity, and behavioral modifications should be the foundation of her weight loss plan. - If pharmacotherapy is considered necessary, medications like Xenical, Qsymia, or Contrave might be more suitable options given her thyroid history. - Consultation with an endocrinologist is advisable to manage her thyroid condition and guide the choice of obesity treatment. Remember, the choice of treatment should be made in consultation with the patient's healthcare provider, taking into account her full medical history, current medications, and individual risk factors.
Claude	Let me help analyze this case carefully and suggest appropriate obesity treatment alternatives, considering the patient's complex medical history and the thyroid nodule situation.<br>Key patient factors to consider: 48-year-old woman - Weight: 80 kg - Existing conditions: Hypertension, hypothyroidism (on Levothyroxine) 8 mm thyroid nodule present - Strong family history of papillary thyroid cancer - Saxenda (liraglutide) is currently contraindicated Given these factors, here are appropriate alternative treatment options: - Other FDA-approved medications:

	• Xenical (orlistat)<br>o Works by blocking fat absorption<br>o Safe with thyroid conditions<br>o Can be used with hypertension medications<br>o Monitor for potential interaction with levothyroxine (separate administration times)<br>• Qsymia (phentermine/topiramate)<br>o Should be used cautiously with hypertension<br>o May need blood pressure monitoring<br>o No known thyroid cancer risks<br>2. Lifestyle interventions:<br>• Structured diet program<br>o Mediterranean diet approach<br>o Calorie restriction (500-750 kcal/day deficit)<br>o Regular meal timing to optimize levothyroxine effectiveness<br>• Exercise program (with medical clearance)<br>o Start with low-impact activities<br>o Gradually increase to 150 minutes/week moderate activity<br>o Include resistance training 2-3 times/week<br>3. Behavioral support:<br>• Cognitive behavioral therapy for weight management<br>• Food journaling<br>• Regular meetings with a registered dietitian

	Important considerations:<br>1. The thyroid nodule should be evaluated (FNA biopsy) before finalizing the weight loss strategy<br>2. Regular monitoring of thyroid function during weight loss<br>3. Coordination between endocrinologist and primary care physician<br>4. Blood pressure monitoring during weight loss intervention

Question 7	
Thyro-GenAI	To assess the characteristics and potential clinical significance of the described thyroid nodules, we can refer to the sonographic ultrasound features and their associated risk of malignancy as outlined in the provided context.<br>Example 1:<br>Nodule Description:<br>o Size: 1.2 x 0.9 x 1.2 cm<br>o Margins: Partially irregular<br>o Appearance: Heterogeneous isoechoic<br>o Rim: Hypoechoic<br>Analysis:<br>1. Margins: Partially irregular margins can suggest a higher suspicion of malignancy. Irregular margins are often associated with invasive growth patterns.<br>2. Appearance: A heterogeneous isoechoic appearance indicates that the nodule has mixed echogenicity, which can be seen in both

benign and malignant nodules.

3. Hypoechoic Rim: This feature can sometimes be associated with a fibrous capsule, which might be seen in benign conditions, but in the context of irregular margins, it could raise suspicion.

Clinical Significance:

- Risk of Malignancy: Based on the irregular margins, this nodule could fall into the "Intermediate suspicion" category, which has a 10%-20% estimated risk of malignancy.
- FNA Recommendation: Given the size (1.2 cm) and the suspicious features, a fine-needle aspiration (FNA) biopsy is recommended for further evaluation.

Example 2:

Nodule Description:

- Size: 7 mm
- Shape: Taller than wide

Analysis:

1. Size: At 7 mm, this nodule is relatively small.
2. Shape: A "taller than wide" shape is a concerning feature often associated with malignancy, as it suggests vertical growth that may invade surrounding tissues.

Clinical Significance:

- Risk of Malignancy: The "taller than wide" shape places this nodule in the "High suspicion" category, with an estimated risk of malignancy of 70%-90%.

	o FNA Recommendation: Despite its small size, the concerning shape may warrant close monitoring or consideration for FNA, especially if there are other risk factors or changes in the nodule's characteristics over time.<br>Conclusion:<br>o Example 1 suggests a moderate risk of malignancy, and FNA is recommended due to its size and irregular margins.<br>o Example 2 has a high suspicion of malignancy due to its shape, and while small, it may require further evaluation or close monitoring.<br>These assessments are based on the guidelines provided by the American Thyroid Association and other relevant sources. Always consult with a healthcare professional for personalized medical advice.
ChatGPT-4	Based on the descriptions provided, the ultrasound findings suggest specific features that radiologists and endocrinologists assess to evaluate thyroid nodules. Here's what each example implies:<br>Example 1:<br>• Size: The nodule measures 1.2 x 0.9 x 1.2 cm.<br>• Margins: The partially irregular margins could be a concern, as irregular or lobulated margins are associated with a higher likelihood of malignancy.<br>• Echogenicity: The nodule is described as heterogeneous isoechoic. Isoechoic means it has a similar echogenicity to the surrounding thyroid tissue, while heterogeneous means it contains

areas of different textures, which may indicate varying types of tissue or changes within the nodule.

- Hypoechoic rim: This suggests the presence of a thin, dark (hypoechoic) border around the nodule, which may indicate a capsule or compressed thyroid tissue. This feature is usually benign but can sometimes be associated with follicular neoplasms.

Interpretation:

- The combination of partially irregular margins and heterogeneous texture may raise suspicion, potentially requiring further evaluation with a fine needle aspiration biopsy (FNAB), especially if other risk factors are present.
- However, since it is isoechoic with a hypoechoic rim, this can sometimes suggest a benign nodule. The overall risk needs to be assessed using a thyroid imaging reporting and data system (TI-RADS) score.

Example 2:

- Size: The nodule is 7 mm.
- Shape: The phrase "taller than wide" refers to the nodule’s shape being more vertical than horizontal on ultrasound. This is an important feature because a taller-than-wide shape is associated with a higher risk of thyroid cancer.
- Comparison with previous study: The change in shape compared

to a prior ultrasound could indicate growth or morphological changes, which might raise concern.

Interpretation:

- A nodule that is taller than wide, even if small (like 7 mm), is considered more suspicious for malignancy, especially if this change is new or has developed since the last imaging study.
- Depending on additional factors such as the presence of microcalcifications, solid composition, or irregular margins, this nodule might also warrant further evaluation with FNAB, especially if the patient has risk factors for thyroid cancer.

Conclusion and Recommendations

- Example 1: The partially irregular margins, heterogeneous texture, and hypoechoic rim suggest mixed features. Depending on clinical context (e.g., family history, previous thyroid issues), a biopsy might be recommended.
- Example 2: A small nodule that is taller than wide should be monitored closely. Given its suspicious shape, further evaluation (potentially including FNAB or follow-up imaging) is often recommended.

In both cases, the specific risk stratification might be done using systems like TI-RADS (Thyroid Imaging Reporting and Data System), which guides the management based on ultrasound features and other risk factors.

Perplexity	Based on the provided descriptions, I'll analyze each example separately:<br><br>Example 1: 1.2 x 0.9 x 1.2 cm thyroid nodule<br><br>This nodule has several characteristics to consider:<br><br>1. Size: The nodule measures 1.2 x 0.9 x 1.2 cm, which is significant enough to warrant further evaluation.<br>2. Margins: Partially irregular margins are described. Irregular margins are an independent predictor for malignancy, with a specificity of 87.1%–98.6% and a positive predictive value of 32.1%–86.7%. This feature increases the suspicion for malignancy.<br>3. Echogenicity: The nodule is described as heterogeneous isoechoic. Isoechoic nodules generally have a lower risk of malignancy (8.6–13.4%) compared to hypoechoic nodules.<br>4. Hypoechoic rim: The presence of a hypoechoic rim or halo is more frequently found in benign nodules. However, it's not highly specific for benignity and can be found in follicular neoplasms as well.<br><br>Implication: This nodule has mixed features. The irregular margins raise concern for potential malignancy, while the isoechoic nature and hypoechoic rim are less suspicious. Given its size and the presence of an irregular margin, this nodule would likely be classified as intermediate to high risk (EU-TIRADS 4 or 5) and would warrant further evaluation with fine-needle aspiration (FNA).

	Example 2: 7 mm thyroid nodule<br>This nodule has the following characteristics:<br>1. Size: The nodule is 7 mm, which is considered small.<br>2. Shape: It is described as "more taller than wide compared to the previous study." A taller-than-wide shape is considered a suspicious ultrasound feature that strongly suggests malignancy.<br><br>Implication: Despite its small size, this nodule exhibits a concerning feature. The taller-than-wide shape is associated with a higher risk of malignancy. This shape change over time is particularly worrisome.<br>In most guidelines, FNA is not routinely recommended for nodules smaller than 1 cm unless there are highly suspicious features or other risk factors. However, the change in shape warrants close monitoring. This nodule would likely be classified as high risk (EU-TIRADS 5) due to its shape.<br>For both examples, it's important to note that while these ultrasound features provide valuable information, they should be considered in conjunction with other clinical factors, such as patient history, physical examination findings, and any relevant laboratory results. The final management decision should be made by a healthcare professional considering all available information.
Claude	Let me analyze these ultrasound descriptions and their implications:<br>Example 1: Size: 1.2 x 0.9 x 1.2 cm Features raising concern:<br>• Partially irregular margins: This can suggest invasive growth

	• Heterogeneous echogenicity: While isoechoic is generally reassuring, heterogeneity can indicate complex internal architecture<br>• Hypoechoic rim (halo): While a complete, thin, regular halo is reassuring, an irregular or thick halo can be concerning<br>Given these characteristics, this nodule would likely be classified as intermediate to high suspicion based on various risk stratification systems (like ACR TI-RADS or ATA guidelines) and would typically warrant fine needle aspiration (FNA) biopsy given its size >1 cm and suspicious features.<br>Example 2: Size: 7 mm Key concerning feature:<br>• Taller-than-wide shape (increased anteroposterior diameter compared to transverse diameter)<br>• Noted change from previous study<br>The taller-than-wide shape is a highly specific feature for malignancy, as benign nodules tend to grow parallel to the tissue plane. Even though this nodule is <1 cm, the presence of this highly suspicious feature might warrant consideration of FNA biopsy or at minimum, close follow-up imaging.

Question 8	
Thyro-GenAI	To determine the recommended next step for a 49-year-old female with a history of a thyroid FNA showing a "favor benign follicular nodule with lymphocytic thyroiditis," and a current ultrasound indicating a

	hypoechoic nodule that has increased in size, we need to consider several factors based on the provided context and guidelines.<br>Step-by-Step Analysis:<br>1. Review of Previous FNA Results:<br>o The previous FNA result was "favor benign," which suggests a low likelihood of malignancy. However, the presence of lymphocytic thyroiditis indicates an underlying inflammatory process, possibly Hashimoto's thyroiditis.<br>2. Current Ultrasound Findings:<br>o The nodule has increased in size from 0.7 cm to 1.2 x 0.9 x 1.2 cm. This represents a significant increase in volume, which is a key factor in deciding further management.<br>o The nodule is described as hypoechoic, which can be a feature associated with malignancy, although it is not definitive on its own.<br>3. Guidelines for Nodule Management:<br>o According to the American Thyroid Association (ATA) guidelines, a significant increase in nodule size (greater than 50% increase in volume) is an indication for repeat FNA sampling.<br>o The increase in size from 0.7 cm to 1.2 cm represents a significant change, warranting further evaluation.<br>4. Recommendation:

	o Repeat FNA: Given the increase in size and the hypoechoic nature of the nodule, a repeat FNA is recommended to reassess the cytological characteristics and rule out any potential malignancy.<br>o Ultrasound Monitoring: Continued ultrasound monitoring may also be advised to track any further changes in the nodule's characteristics.<br>Conclusion:<br>The recommended next step for this patient is to perform a repeat fine needle aspiration (FNA) to evaluate the nodule's current cytological status, given the significant increase in size and the hypoechoic ultrasound appearance. This approach aligns with the guidelines for managing thyroid nodules with significant growth and potentially suspicious features.
ChatGPT-4	Given the history and current findings, the next step should be repeat fine needle aspiration (FNA) of the thyroid nodule. Here's the reasoning:<br>Clinical Context:<br>1. Previous FNA Result: Five years ago, the FNA result indicated a benign follicular nodule with lymphocytic thyroiditis. However, there is always a small risk of false negatives with initial FNA results.<br>2. Growth in Nodule Size: The current ultrasound indicates that the nodule has grown from 0.7 cm to 1.2 cm in the last five years. An increase in size is concerning, particularly in the context of a

	hypoechoic nodule, which is more likely to be associated with malignancy compared to other echogenicities.<br>3. Hypoechoic Appearance: Hypoechoic nodules on ultrasound are often more suspicious for malignancy, especially when combined with growth in size over time.<br>Recommendation:<br>According to the American Thyroid Association (ATA) guidelines, the criteria for repeat FNA include:<br>• A nodule that has grown by more than 20% in at least two dimensions and has increased by at least 2 mm.<br>• Suspicious ultrasound features such as hypoechogenicity.<br>In this case:<br>• The nodule has grown by more than 5 mm in diameter and shows a hypoechoic pattern.<br>• Given the changes, the most appropriate next step is a repeat FNA to rule out malignancy.<br>Summary of the Next Step:<br>• Order a repeat FNA biopsy of the thyroid nodule.<br>• Further management will depend on the biopsy results. If the repeat FNA confirms benign features, continued monitoring with periodic ultrasound may be sufficient. However, if suspicious or malignant features are found, additional steps like surgery may be warranted.
Perplexity	Based on the information provided, the next step in managing this

patient's thyroid nodule should be as follows:

Evaluation and Management

The patient's thyroid nodule requires further evaluation due to its significant growth over the past 5 years. Here's a breakdown of the key factors and recommended next steps:

Growth Assessment

The nodule has increased from 0.7 cm to 1.2 cm in its largest dimension, which represents a growth of more than 70%. This growth is clinically significant, as it exceeds the threshold for concern established by multiple guidelines:

- An increase of more than 50% in nodule volume or 20% in at least two nodule dimensions is considered clinically significant.
- The American Thyroid Association recommends further evaluation for nodules with >20% growth in at least two dimensions or a >50% increase in volume.

Recommended Next Step

Given the significant growth of the nodule, the recommended next step is to perform a repeat fine needle aspiration (FNA) biopsy. This is necessary because:

1. The previous FNA was performed 5 years ago, which is a considerable time interval.
2. The nodule has shown significant growth, which raises the possibility of malignancy despite the previous benign cytology.
3. The current size of 1.2 cm meets the general threshold for biopsy

recommendation in most thyroid imaging reporting systems.

Additional Considerations

- Ultrasound characteristics: The nodule is described as hypoechoic, which can be a feature associated with higher risk of malignancy. However, a complete risk stratification would require additional ultrasound features (e.g., margins, echogenic foci, shape).
- Patient age: At 49 years old, the patient is in an age group where thyroid nodules should be evaluated thoroughly, as the risk of malignancy can increase with age.
- Previous diagnosis: While the previous FNA result favored a benign diagnosis, it's important to note that benign nodules can occasionally transform or be misdiagnosed initially.

Follow-up Plan

After the repeat FNA biopsy:

1. If the result is benign, consider follow-up ultrasound in 12-24 months.
2. If the result is indeterminate or suspicious, further management may include molecular testing or surgical consultation based on the specific cytology category.
3. Regardless of the FNA result, continued ultrasound surveillance will be important due to the nodule's growth history.

	By following these steps, the healthcare provider can ensure appropriate evaluation and management of this growing thyroid nodule, balancing the need for cancer detection with avoiding unnecessary procedures.
Claude	Let me think about this case step by step:<br>1. Key points to consider:<br>• Previous FNA was benign (favor follicular nodule with lymphocytic thyroiditis)<br>• Current nodule is 1.2 cm in largest dimension<br>• Has grown from 0.7 cm over 5 years<br>• Nodule is hypoechoic on ultrasound<br>2. Relevant factors:<br>• Growth of >50% in volume or a 20% increase in at least two dimensions qualifies as significant nodule growth<br>• In this case, the nodule has grown from 0.7 cm to 1.2 cm (>70% increase)<br>• Hypoechoic appearance is a suspicious sonographic feature<br>3. Guidelines to consider:<br>• According to ATA guidelines, nodules with prior benign FNA that show significant growth should undergo repeat FNA<br>• The growth pattern here raises concern for potential malignancy despite previous benign FNA<br>4. Decision-making:<br>• The significant growth and sonographic features warrant further evaluation

	• Previous benign FNA from 5 years ago cannot definitively exclude current malignancy<br>Based on these factors, the next step should be to perform a repeat FNA biopsy of the nodule. This is indicated due to:<br>1. Significant documented growth<br>2. Suspicious ultrasound feature (hypoechoic)<br>3. Time elapsed since previous FNA (5 years)

Question 9	
Thyro-GenAI	When considering the types of cancers a 57-year-old female with a history of papillary thyroid cancer should be cautious about, it's important to take into account several factors, including the nature of papillary thyroid cancer, potential genetic predispositions, and general cancer risk factors associated with age and gender. Here's a detailed breakdown:<br>1. Recurrence of Thyroid Cancer<br>o Papillary Thyroid Cancer (PTC) Recurrence: Although PTC generally has a good prognosis, there is a risk of recurrence, particularly in the cervical lymph nodes. Regular follow-ups with ultrasound and thyroglobulin tests are recommended.<br>2. Other Thyroid Cancers<br>o Follicular Thyroid Cancer: While less common, there is a possibility of developing other types of thyroid cancer. Monitoring for any new thyroid nodules is essential.<br>3. Secondary Cancers

	o Breast Cancer: Women with a history of thyroid cancer may have a slightly increased risk of breast cancer. Regular mammograms and breast exams are advised.<br>o Colorectal Cancer: Age is a risk factor for colorectal cancer, and regular screenings such as colonoscopies are recommended starting at age 50, or earlier if there is a family history.<br>4. Genetic Syndromes<br>o Familial Cancer Syndromes: If there is a family history of multiple types of cancer, genetic counseling might be beneficial to assess the risk of syndromes like Cowden syndrome, which can increase the risk of breast, thyroid, and other cancers.<br>5. General Cancer Risks<br>o Lung Cancer: Smoking history, if applicable, increases the risk of lung cancer. Smoking cessation and regular screenings are important.<br>o Gynecological Cancers: Regular pelvic exams and Pap smears are important for early detection of cervical and ovarian cancers.<br>6. Lifestyle and Environmental Factors<br>o Diet and Exercise: Maintaining a healthy lifestyle with a balanced diet and regular exercise can help reduce the risk of various cancers.<br>o Radiation Exposure: Previous radiation treatments, especially in the neck area, can increase the risk of other cancers. Discussing past treatments with a healthcare provider is crucial for risk

	assessment.<br>Conclusion<br>For a 57-year-old female with a history of papillary thyroid cancer, vigilance in monitoring for recurrence, regular screenings for other common cancers, and consideration of genetic factors are key components of a comprehensive cancer prevention strategy. Regular consultations with healthcare providers for personalized risk assessments and screening schedules are highly recommended.
ChatGPT-4	Given her history of papillary thyroid cancer, a 57-year-old female should be particularly vigilant about several other types of cancers. While papillary thyroid cancer is generally associated with a good prognosis, there are increased risks for certain other cancers due to factors such as shared genetic predispositions, treatment side effects, and underlying risk factors.<br>1. Other Thyroid Cancers<br>• Follicular thyroid cancer or anaplastic thyroid cancer can occasionally develop in patients with a history of papillary thyroid cancer.<br>• Medullary thyroid cancer is less common but can also coexist, especially if there is a genetic component (e.g., Multiple Endocrine Neoplasia Type 2).<br>2. Breast Cancer<br>• Studies have shown that women with a history of papillary thyroid cancer have a slightly increased risk of developing breast

cancer, possibly due to hormonal factors or shared genetic mutations (like BRCA mutations).

- Regular mammograms and breast examinations are advised.

3. Skin Cancer (Melanoma)

- There is a known association between thyroid cancer and melanoma. Increased vigilance in monitoring for suspicious skin lesions is warranted.

4. Colorectal Cancer

- Patients with a history of thyroid cancer, particularly those treated with radiation, may have a slightly increased risk of developing colorectal cancer.
- Routine colonoscopy screening is recommended, especially after the age of 50.

5. Lymphoma

- Rarely, thyroid lymphoma may develop, particularly in patients with a history of autoimmune thyroid diseases like Hashimoto’s thyroiditis.

6. Uterine (Endometrial) and Ovarian Cancer

- While the link is not as strong, some studies suggest a slight increase in endometrial and ovarian cancer risk, potentially due to shared hormonal risk factors.

7. Esophageal Cancer

- There is a minor increased risk of esophageal cancer among thyroid cancer survivors, possibly related to previous radiation

treatment affecting nearby tissues.

Factors Contributing to Increased Cancer Risks

- Genetic Syndromes: Conditions like Cowden syndrome and Multiple Endocrine Neoplasia (MEN) can increase the risk of multiple cancers, including thyroid, breast, and colorectal cancers.
- Radiation Exposure: Patients treated with radiation therapy (especially to the neck area) are at increased risk for head and neck cancers, salivary gland tumors, and other malignancies.
- Hormonal Influence: The link between thyroid and breast cancer suggests that hormonal imbalances might play a role in increasing the risk for hormone-sensitive cancers.

Recommended Surveillance and Preventive Measures

1. Regular follow-up with an oncologist or endocrinologist.
2. Breast cancer screening: Annual mammograms and breast self-exams.
3. Skin checks: Regular dermatological evaluations for melanoma.
4. Colonoscopy: Every 10 years (or more frequently if indicated) to screen for colorectal cancer.
5. Routine pelvic exams: To monitor for uterine or ovarian cancer.
6. Lifestyle modifications: Healthy diet, regular exercise, and avoiding smoking can reduce overall cancer risk.

Understanding the specific risk profile of the patient and having a

	personalized cancer surveillance plan is crucial to early detection and improved outcomes.
Perplexity	Given the patient's history of papillary thyroid cancer, there are several types of cancers she should be particularly cautious about:<br>Increased Risk of Second Cancers<br>Survivors of thyroid cancer, including papillary thyroid cancer, have an increased risk of developing certain types of second cancers. For this 57-year-old female patient, the following cancers warrant special attention:<br>Breast Cancer<br>Women who have had thyroid cancer have an elevated risk of developing breast cancer. Regular breast cancer screenings and mammograms are particularly important for this patient.<br>Kidney Cancer<br>There is an increased risk of kidney cancer among thyroid cancer survivors. The patient should be aware of any symptoms related to kidney function and discuss appropriate screening methods with her doctor.<br>Adrenal Cancer<br>Thyroid cancer survivors have a higher risk of developing adrenal cancer. While adrenal cancer is rare, the patient should be vigilant about any symptoms that could indicate adrenal gland issues.<br>Other Potential Risks<br>Depending on the treatment received for her papillary thyroid cancer, the patient may also be at risk for:

	1. Acute Lymphocytic Leukemia (ALL)<br>2. Stomach cancer<br>3. Salivary gland cancer<br>These risks are particularly elevated if the patient received radioactive iodine treatment.<br>Additional Considerations<br>It's important to note that the risk of second cancers can vary based on factors such as the specific treatment received and the time elapsed since the initial thyroid cancer diagnosis. Studies have shown that the average interval between thyroid cancer and a second primary cancer is about 6 years.<br>Recommendations<br>To manage these increased risks, the patient should:<br>1. Attend regular follow-up appointments with her healthcare provider.<br>2. Undergo recommended cancer screenings appropriate for her age and risk factors.<br>3. Be aware of and report any new or unusual symptoms promptly.<br>4. Maintain a healthy lifestyle, including a balanced diet, regular exercise, and avoiding tobacco use.<br><br>While these increased risks are concerning, it's important to remember that many thyroid cancer survivors do not develop second cancers. The patient should work closely with her healthcare team to develop an

	appropriate monitoring and prevention plan tailored to her individual needs and medical history.
Claude	Given a history of papillary thyroid cancer (PTC), there are several important cancer risks to consider for this patient:<br>1. Primary Concerns:<br>• Recurrence of papillary thyroid cancer - requires ongoing monitoring<br>• Second primary thyroid cancer of a different histological type<br>• Local or regional metastases in neck lymph nodes<br>2. Associated Cancer Risks:<br>• Breast cancer - There is evidence of an increased risk of breast cancer in women with a history of thyroid cancer<br>• Renal cell carcinoma - Some studies have shown an association between PTC and kidney cancer<br>• Salivary gland tumors - There appears to be a modest increased risk<br>3. Genetic Considerations: If the patient's PTC was related to certain genetic syndromes, additional cancer risks may include:<br>• Familial Adenomatous Polyposis (FAP): increased risk of colorectal cancer<br>• Cowden syndrome: increased risk of breast, uterine, and other cancers<br>• DICER1 syndrome: increased risk of various rare tumors<br>Recommendations:

	• Regular thyroid cancer surveillance<br>• Age-appropriate cancer screening (mammography, colonoscopy)<br>• Consider genetic counseling if there are concerning features in personal or family history<br>• Maintain awareness of any new symptoms or concerns
