# Supplementary material for: Thyro-GenAI: A Chatbot Using Retrieval-Augmented Generative Models for Personalized Thyroid Disease Management
Source: J Clin Med. 2025 Apr 3;14(7):2450. doi: 10.3390/jcm14072450 (PMC11989359; doi:10.3390/jcm14072450)
Supplement: Supplementary file 1 [file jcm-14-02450-s001.zip › Supplementary Material S3.pdf]

Supplemental Data S3. Free text evaluation

Supplemental Data S3-1. Free text evaluation for the quality of responses

Question Number	Thyro-GenAI	ChatGPT-4	Perplexity	Claude
1	Not specific.<br><br>Aligns with guidelines, but thyroid antibody testing is omitted.<br><br>Minimal unnecessary information.	Described in detail but missing some key information.	Most specific and accurate, included guidelines.<br><br>Incorrect Levothyroxine start criteria, pregnancy TSH range, and FNA indications.<br><br>Includes unnecessary information.	Concisely summarized including key content.<br><br>Relatively contains little information.<br><br>Includes inaccurate contents.
2			Most specific and well-cited	Short summary but lacks consensus.

3	Contains a lot of content but lacks key information			Provided risk evaluation for invasive procedures and explained corresponding countermeasures
4	Most specific and described follow-up steps based on biopsy results.<br><br>Most accurate understanding and described the next steps based on biopsy results.		Specified follow-up period	
5	Did not specify a follow-up period			

6	Discussed bariatric surgery but did not mention other medication side effects<br><br>The question was about the obesity treatment option, but offred information about thyroid nodules as top priority, weak on Obesity-related information<br><br>Nevertheless, the benefits of addressing all aspects in an integrated way	Discussed bariatric surgery and explained medication side effects	Mentioned indications for bariatric surgery, suggested lifestyle modifications as first-line treatment, presented the most diverse range of medications but some are not suitable as diet pills	Provided the least information

7		Has the best readability		Written in a format understandable to experts but difficult for general doctors or patients
8	Provided the most appropriate answer and best data supporting treatment.			The inference was accurate, but the response only addressed the diagnosis (treatment X).
9		Presented most of the syndromes related to familial NMTC and thyroid cancer.		Suggested inappropriate tests for breast cancer screening and recommended somewhat excessive screenings

Supplemental Data S3-2. Free text evaluation for the quality of references used

Question Number	Thyro-GenAI	ChatGPT-4	Perplexity	Claude
1	Not specific but provided the most appropriate resources<br><br>Only the nodule section was cited from the 2017 ATA Pregnancy Thyroid Guidelines, but it is necessary to also include the part about thyroid dysfunction (subclinical hypothyroidism)<br><br>It would be beneficial to present the latest domestic	Did not provide information	Focused on latest research but had less reliable sources compared to Thyro-GenAI.<br><br>Had a problem that mainly included unauthorized sources.	Did not provide information

	guidelines (e.g., 2023 pregnancy recommendations, 2023/2024 nodule recommendations).			
2	Responded correctly but displayed an error message<br><br>Although it cites authoritative sources (textbooks, guidelines), the cited content often does not support the response.	Both GPT and Claude did not provide citations; GPT provided more information		
3	The *Sabiston, Surgery of the Thyroid and Parathyroid Glands* -> textbook tends to be mainly a surgical			

	reference.<br><br>The MTC guideline was incorrectly cited as a reference.<br><br>Updates to the latest domestic guidelines (2023/2024) are needed.			
4			Provided fewer citations than usual and did not properly reference related literature<br><br>Incorporation of the latest 2023 KTA Thyroid Nodule Guidelines	

5	There are references with low relevance (e.g., regarding MTC content).			
6	Citations are good but lacks sufficient information on side effects<br><br>There is no reference or guideline related to obesity.			
7	Used the most reliable citations and presented them in an easy-to-read format.<br><br>Extracting information from the ATA pregnancy guideline was			

	inappropriate, as there is no indication that the case involved a pregnant patient.			
8	Provided the most appropriate citations.<br><br>The MTC guideline and pediatric thyroid nodule/cancer guidelines were cited as references, but they do not support the response.<br><br>Parts cited from the *Surgery of the Thyroid and Parathyroid Glands* textbook			No citations provided

	(e.g., RFA/Laser, MTC) were not relevant.<br><br>The KTA thyroid nodule guideline is not the latest recommendation, and the cited content does not support the response.<br><br>It would be better if the first/last words of cited phrases were not cut off. For example, "staining to eval" instead of "evaluate" being truncated to "eval."			

9	Shorter response compared to other AIs but provided the most appropriate citations and data			
